# Stage-Resolved Dynamics of Antibiotic Resistance Genes and Mobile Genetic Elements in Full-Scale Anaerobic Digestion Systems

**DOI:** 10.1021/acs.est.6c04399

**Published:** 2026-10-31

**Authors:** He Sun, Sara B. Nilsson, Anna Schnürer

**Affiliations:** † Department of Molecular Sciences, 8095 Swedish University of Agricultural Sciences, Uppsala 75007, Sweden; ‡ Hushållningssällskapet, Eldsberga, Halland 30596, Sweden

**Keywords:** Antibiotic resistance, antibiotic resistance genes, mobile genetic elements, anaerobic digestion, biogas plants, full scale, digestate

## Abstract

Anaerobic digestion (AD) has the potential to reduce antibiotic resistance genes (ARGs) and mobile genetic elements (MGEs) during waste treatment, yet evidence from full-scale systems remains limited. In particular, process-stage dynamics and host-associated resistance mechanisms are poorly understood, and the relative importance of operational and physicochemical drivers remains unresolved. Here, we profiled process-oriented, stage-resolved resistome and mobilome dynamics across substrates, digestates, and storages from ten farm-scale AD plants, resolving putative host-ARG-MGE coabundance associations across sequential process stages. An integrated framework combining high-throughput qPCR and 16S rRNA gene sequencing was used to quantify ARG and MGE dynamics and characterize microbial community structure. Across different AD systems, ARGs and MGEs exhibited heterogeneous abundance changes, with no consistent patterns. However, elevated pH and NH_3_ were associated with decreasing trends but were not solely determinative. Stage-resolved correlation networks revealed that ARG-MGE associations formed a conserved structural backbone, whereas putative host associations underwent continuous reshuffling across process stages. This indicates that resistance persistence is driven less by stable taxon associations and more by the redistribution of ARG-MGE units among microbial taxa. Collectively, these results demonstrate that stage-resolved, network-informed analyses provide an informative framework for evaluating resistance persistence and potential environmental risks associated with digestate application.

## Graphical abstract

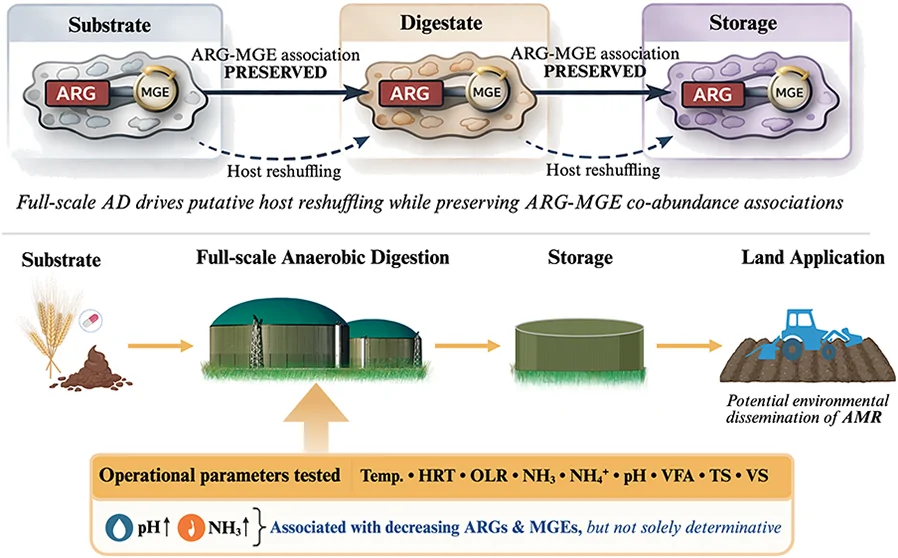

## Introduction

Anaerobic digestion (AD) is a key component of circular agricultural systems, as it enables the conversion of organic manure and crop residues into renewable energy (biogas) and nutrient-rich digestates.^1^ Digestates derived from AD systems are widely applied as organic fertilizers due to their high availability of plant nutrients, which contributes to fertilization efficiencies comparable to mineral fertilizers.^2^ In addition, biogas production contributes to climate mitigation by reducing greenhouse gas emissions and improving on-farm energy balance.^3^ As a result, the large-scale reuse of digestates on agricultural land has increased substantially across Europe in recent years.^4^ Given the central role of digestates in nutrient recycling within circular agriculture, ensuring their safe application at scale is essential to prevent the release of chemical or biological contaminants into the environment.

Among the potential risks associated with digestate application, growing attention has been directed toward the unintended dissemination of antibiotic resistance. Antibiotic resistance is widely recognized as a major global health challenge spanning human, animal, and environmental domains.^5,6^ Digestates originating from livestock manure, food waste, and other organic substrates have been shown to contain antibiotic resistance genes (ARGs) and mobile genetic elements (MGEs),^7–9^ which together determine both the persistence and mobility of resistance in environmental systems. Although ARGs are largely introduced via incoming substrates rather than generated during digestion,^7,10^ their fate during AD remains a central concern for assessing downstream environmental risks.

Previous studies have reported both reductions and enrichments of ARGs during AD, with outcomes varying widely across systems.^10–14^ Such variability partly reflects gene-specific responses, whereby certain ARGs may be enriched even when the majority of resistance genes are reduced under the same conditions.^15^ In addition, the extent of ARG reduction is influenced by operational and physicochemical parameters, including substrate composition, pretreatment, temperature, retention time, organic loading rate, and downstream handling practices.^7,10^ Thermophilic AD has often been associated with greater ARG reduction than mesophilic operation,^11,16,17^ and multistage systems or high-solids AD have in some cases outperformed single-stage or wet-type systems.^18–21^ However, most existing studies remain laboratory-based. Although several full-scale studies have examined ARG fate and potential operational drivers, they are still constrained by limited numbers of biogas plants and relatively narrow ARG target panels,^18,22,23^ limiting their ability to capture general patterns across full-scale biogas systems and comprehensive ARG dynamics under real-world operating conditions. Therefore, systematic and process-oriented investigations remain limited, especially those comparing multiple full-scale agricultural biogas plants and linking operational and physicochemical conditions to ARG dynamics across sequential AD stages.

Beyond abundance-based assessments, increasing attention has been directed toward host-ARG and ARG-MGE associations, as ARGs can persist within microbial hosts via vertical gene transfer and disseminate among bacteria through horizontal gene transfer mediated by MGEs.^11,24^ Correlation-based network analyses have therefore been widely applied to infer putative host-ARG and ARG-MGE relationships, providing insights into potential resistance carriers and mobility pathways during AD.^21,25^ However, most existing studies construct such networks from aggregated samples by pooling substrates and digestates,^11,19,21,25^ thereby providing static snapshots of host-ARG-MGE associations. Although previous studies have provided valuable insights into ARG, MGE, and microbial community dynamics during AD, limitations related to pooled sampling designs, restricted ARG/MGE panels, and laboratory-scale systems have prevented systematic resolution of host-ARG-MGE associations across sequential stages of full-scale AD systems. This limits our understanding of whether resistance is primarily reduced or redistributed during the digestion process.

In this study, this knowledge gap is assessed by applying a stage-resolved framework to investigate ARG and MGE dynamics across full-scale AD and postdigestion storage systems. The main methodological advance of this study lies not in profiling full-scale systems alone, but in resolving how putative host-ARG-MGE associations are reorganized across sequential process stages under real-world operating conditions. Samples were collected from ten farm-scale biogas plants in Sweden, representing organic, conventional, and mixed farming practices. An integrated approach combining high-throughput quantitative PCR (qPCR) targeting ARGs and MGEs, 16S rRNA gene amplicon sequencing, and 16S rRNA gene qPCR was applied to characterize the resistome, mobilome, and bacterial community structure and abundance. This framework enables the investigation of how operational and physicochemical parameters may influence resistance dynamics, as well as how ARGs are redistributed among putative microbial hosts and MGEs along the AD process. Together, this study provides a process-resolved understanding of antibiotic resistance dynamics in full-scale biogas systems, with direct implications for digester operation, postdigestion storage, and the safe agricultural reuse of digestates.

## Materials and Methods

### Samples and Biogas Plant Operations

A total of 42 samples were collected from ten full-scale biogas plants located across central and southern Sweden (Table S1). The biogas plants were classified according to substrate origin into organic (*n* = 3), conventional (*n* = 3), and mixed conventional-organic systems (*n* = 4). Plant operating conditions and sample characteristics are summarized in Table S2. All samples were stored at −20 °C prior to DNA extraction and physicochemical analyses. Because this study was conducted in full-scale operating biogas plants, the sampling design was observational and did not include independent biological replicates for each plant or process stage.

### Physicochemical and Molecular Analyses

Physicochemical analyses, including total solids (TS), volatile solids (VS), pH, ammonium nitrogen (NH_4_^+^-N), ammonia nitrogen (NH_3_–N) and volatile fatty acids (VFAs), as well as DNA extraction and purification procedures, are described in the Supporting Information.

Relative abundances (RA) of ARGs and MGEs were determined by Resistomap Oy (Helsinki, Finland) using customized SmartChip qPCR primer sets. The panel included 323 ARG subtypes and 52 MGE markers (Table S3), with assay naming based on the CARD database, quality-control criteria, and normalization procedures described in the Supporting Information. Because this approach targets a predefined gene panel, the reported resistome and mobilome profiles should be interpreted as panel-defined ARG and MGE dynamics rather than a complete inventory of all resistance and mobility determinants present in the samples.

Absolute abundances (AA) of total bacteria were quantified by 16S rRNA gene-targeted qPCR using primers Bac338F and Bac534R on a QuantStudio 5 Real-Time PCR System with SYBR Green chemistry. Microbial community composition was characterized by 16S rRNA gene amplicon sequencing on the Illumina platform by Novogene (Netherlands), following a previously described protocol.^26^ Details of qPCR conditions, quality-control criteria, and sequencing procedures are described in the Supporting Information.

### Absolute Abundance Calculation for ARGs and MGEs

AA of ARGs and MGEs, expressed as gene copy numbers per gram of wet mass, were calculated by integrating relative gene abundances obtained from ResistoMap with absolute 16S rRNA gene copy numbers quantified by qPCR and DNA yields measured using a Qubit fluorometer. The calculation workflow for AA estimation is provided in Figure S1. For substrate mixtures composed of multiple input materials, AA values were calculated as the mass-weighted sum of the AA of the individual substrates. RA of substrate mixtures were subsequently derived by normalizing the AA values to corresponding 16S rRNA gene abundances within the same mixtures.

### Multivariate and Ordination Analyses

Amplicon sequencing data of the 16S rRNA gene were processed using DADA2 (version 1.16) following established pipelines.^27^ Nonmetric multidimensional scaling (NMDS) was used to visualize similarities among biogas digesters based on physicochemical characteristics and microbial community composition. NMDS of physicochemical parameters was performed using Euclidean distance, while NMDS of microbial community composition was conducted using Bray–Curtis dissimilarity. All ordinations were performed with two dimensions, and a fixed random seed was used to ensure reproducibility.

### Network Analysis of Putative Host-ARG-MGE Associations

Prior to network construction, feature filtering was applied to reduce sparsity and multiple-testing burden. ARGs, MGEs, and bacterial genera were retained if detected in at least 30% of samples (≥13/42), whereas phyla were retained if detected in at least 10% of samples (≥5/42). A lower prevalence threshold was used for phyla because phylum-level features were fewer and represented broader taxonomic groups than genus-level features. To further improve statistical power, the analysis was restricted to the top 50 bacterial genera ranked by mean RA and the top 80 ARG subtypes ranked by total RA across all samples. Phylum-level taxa and MGEs were not further reduced by abundance because their feature numbers were already limited after prevalence filtering.

Before CLR transformation, zero values were replaced by adding half of the minimum nonzero abundance within each filtered feature table. The resulting relative abundance tables were CLR-transformed prior to correlation analysis, and features with zero variance after transformation were removed.^28^ Multiple testing was controlled using false discovery rate (FDR) correction.^29^ For stage-wise association dynamics, Spearman correlation analysis (|ρ| ≥ 0.6, FDR-adjusted *p* < 0.05) was first applied to CLR-transformed data across all samples to identify significant genus-ARG, genus-MGE, and ARG-MGE associations. These global associations were then evaluated within individual process stages using a slightly relaxed but still conservative threshold (|ρ| ≥ 0.5, FDR-adjusted *p* < 0.05), allowing assessment of retained, lost, or newly supported associations without reconstructing independent networks from small stage-specific subsets. In addition, conventional tripartite networks linking phylum-level taxa, ARGs, and MGEs were constructed using Spearman correlation (|ρ| ≥ 0.6, FDR-adjusted *p* < 0.05) and visualized in Gephi (version 0.10.1).

### Statistical Analysis of Directional Shifts in ARGs and MGEs

Changes in the RA of ARGs and MGEs were quantified for each biogas plant as Δ = (downstream process stage −upstream stage: Digestate −Substrate; Storage −Digestate). For each biogas plant, Δvalues were first calculated at the subtype level of ARG and MGE. For class-level analyses, subtype-level Δvalues were aggregated within each plant by calculating the median for each ARG class or MGE category. These class-level Δvalues were then evaluated across plants using two-sided Wilcoxon signed-rank tests against zero. For overall ARG and MGE analyses, subtype-level Δvalues were further aggregated within each plant by calculating the median across all subtypes prior to statistical testing. Missing Δvalues, corresponding to resistance determinants not detected at a given process stage within a system, were excluded on a per-comparison basis. All analyses were performed in R. Given the observational design of full-scale biogas plant survey, statistical analyses were restricted to evaluating directional trends in ARG and MGE abundances across digesters, rather than inferring absolute removal or enrichment.

## Results

### Changes in Absolute and Relative Abundances of ARGs and MGEs across Anaerobic Digestion Systems

Across all substrate mixes, the absolute abundance (AA) of ARGs ranged from 3.44 × 10^8^ to 2.07 × 10^10^ gene copies per gram of wet mass, while MGEs ranged from 5.39 × 10^8^ to 1.03 × 10^10^ gene copies per gram of wet mass. No significant differences in the AA of ARGs or MGEs were observed among substrate mixes derived from different farming practices (Figure 1A). In contrast, changes in AA throughout the AD system varied substantially among the different biogas plants, with both increases and decreases observed (Figure 1B). During AD, decreases in AA were observed in plants H1, K2, and S, whereas increases occurred in plants L, V, and J (Table 1). During postdigestion storage, reductions in AA were detected in plants K1, K2, and N, while increases were observed in plants L, V, J, and H2 (Table 1). It should be noted that, while such changes may reflect genuine biological enrichment driven by microbial proliferation, exceptionally large percentage changes in AA (e.g., + 2042% in V_D2 and +1848% in L_D) should be interpreted cautiously, as differences between substrate and digestate matrices may affect DNA extraction efficiency, while potential residual PCR inhibition may influence qPCR-based quantification.

**Figure 1:**
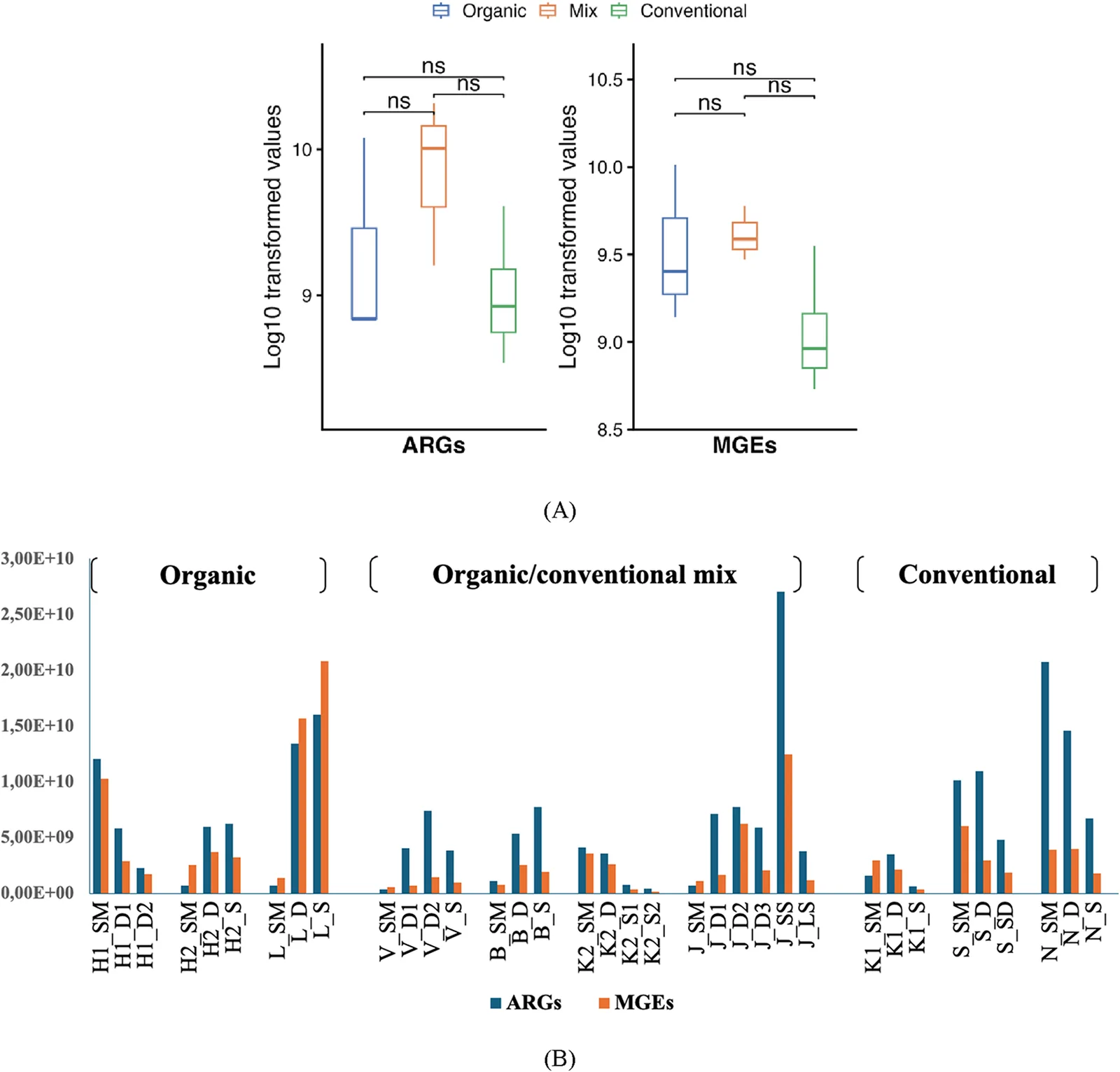
Absolute abundance of antibiotic resistance genes (ARGs) and mobile genetic elements (MGEs), expressed as gene copy numbers per gram of wet mass. (A) Abundance variation of ARGs and MGEs in substrates grouped by farming practices (organic, conventional, and mixed). Pairwise differences among farming practices were evaluated using two-sided Wilcoxon rank-sum tests. (B) Abundance dynamics across anaerobic digestion and postdigestion storage in ten full-scale biogas plants. Each bar in panel B represents one process-stage sample from a full-scale biogas plant. Error bars are not shown because independent biological replicates were not available.

**Table 1:**
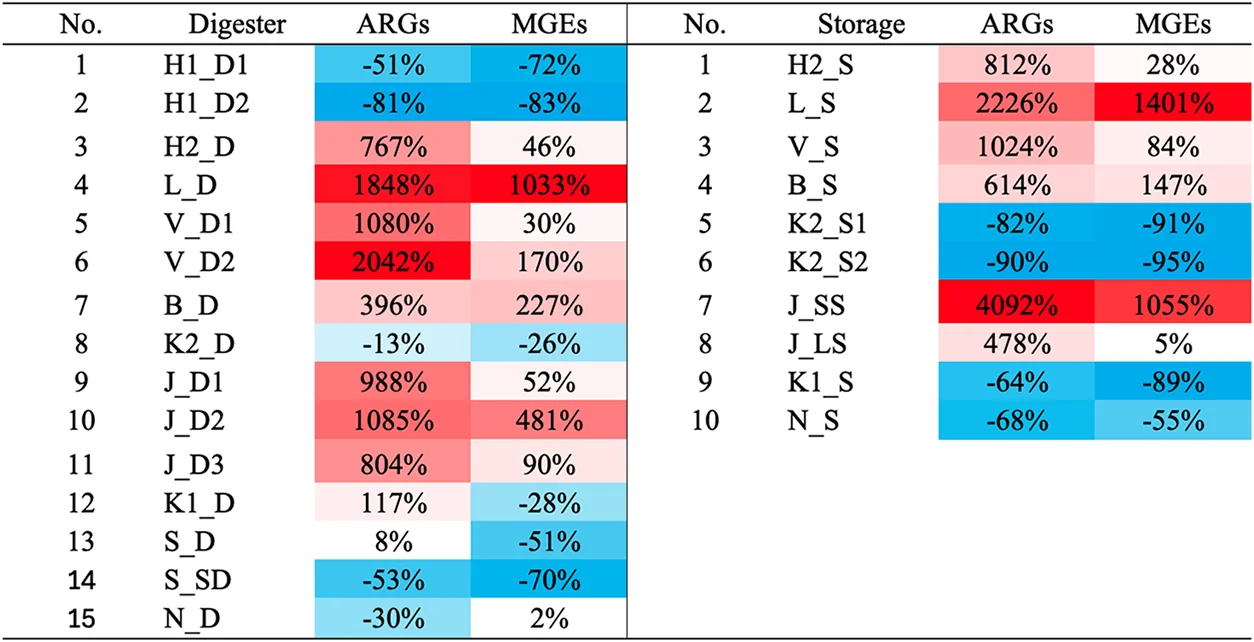
Percentage Changes in Absolute Abundances of Antibiotic Resistance Genes (ARGs) and Mobile Genetic Elements (MGEs) during Anaerobic Digestion (Digester) and Post-Digestion Storage (Storage), Calculated Relative to the Corresponding Substrates (Digester–Substrate; Storage–Substrate)^a^

a Absolute abundances refer to gene copy numbers per gram of wet mass.

Changes in the relative abundance (RA) of ARG subtypes also exhibited heterogeneous responses to AD, with both positive and negative shifts observed across antibiotic classes and digesters (Figure 2A). Compared to substrate, none of the ARG classes showed a statistically significant directional shift in RA across the 15 digesters (FDR-adjusted *p* > 0.05 for all classes), indicating that no specific ARG class was more likely to be enriched or reduced during AD. When RA changes across all ARG subtypes were aggregated, digesters exhibiting negative and positive Δ*v*alues generally corresponded to those showing decreasing and increasing trends in AA, respectively. However, when examined across all 15 digesters as a whole, the median change in RA did not deviate from zero (median Δ = 5.38 × 10^–6^, FDR-adjusted *p* = 0.798), indicating absence of a consistent directional shift in ARG RA during full-scale AD. Together, these results indicate that ARG dynamics during full-scale AD were strongly system-specific rather than showing a uniform response to AD. This system-specific variability was further interpreted in relation to operational and physicochemical conditions in the subsequent analysis and discussion of driver associations and system configurations.

**Figure 2:**
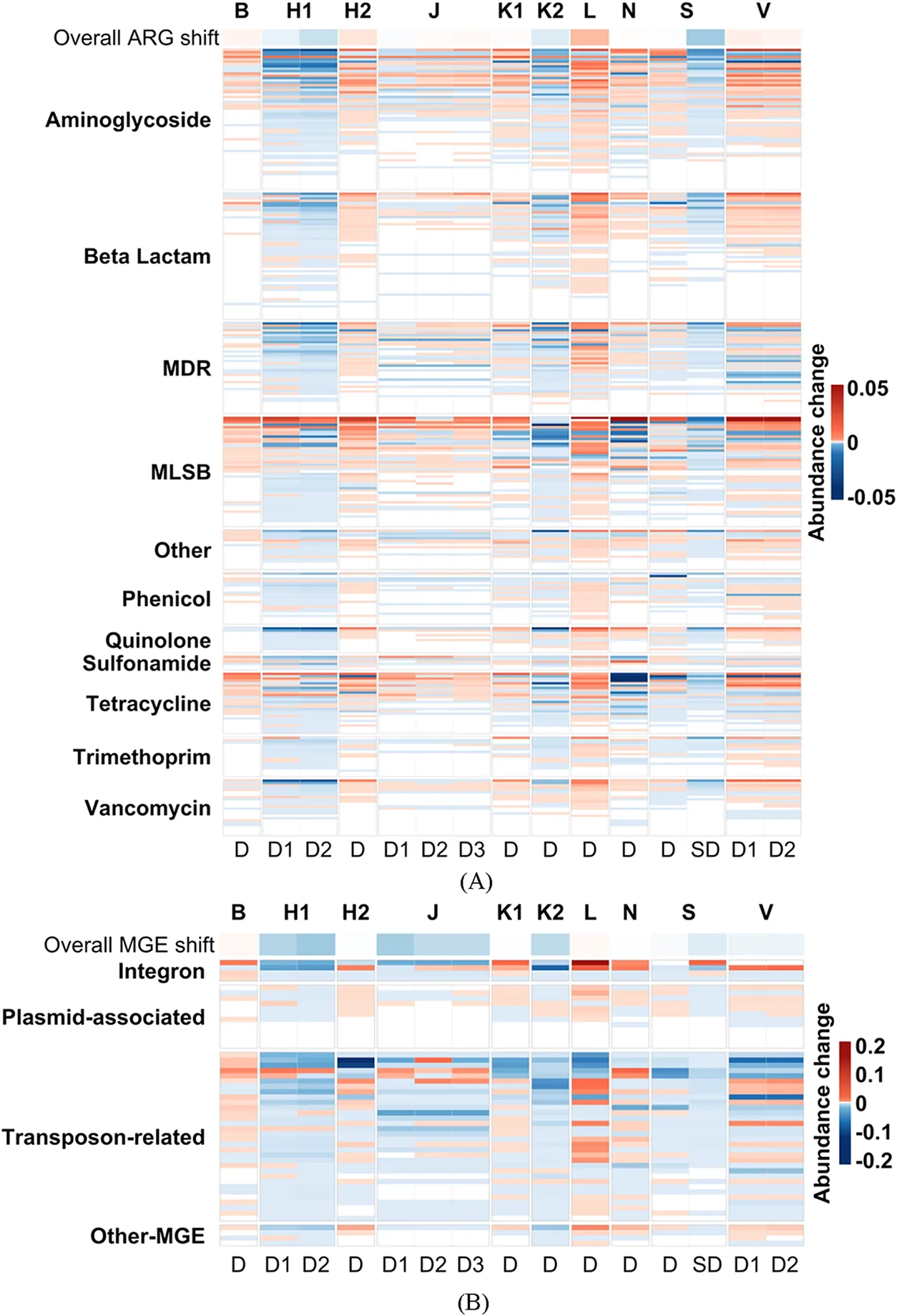
Fate of antibiotic resistance genes (ARGs) and mobile genetic elements (MGEs) during anaerobic digestion in ten full-scale biogas plants. Changes in relative abundance are expressed as the difference between digester and corresponding substrate samples (digester – substrate; for the digester S_SD, values are expressed as SD – D). (A) ARG subtypes grouped by antibiotic classes. (B) MGEs grouped by functional categories. Columns represent individual digesters; D and SD denote digester and secondary digester, respectively. For both panels, the top bar summarizes the overall median change (Δ) in relative abundance for each digester, where blue and red indicate negative and positive deviations, respectively. The color scale of the top bar is independent from that of the main heatmap and is used solely to indicate the direction of overall change.

In contrast to ARGs, distinct patterns were observed for RA changes of MGEs during AD (Figure 2B). Transposon-related MGEs exhibited a statistically significant negative directional shift in RA across digesters (median Δ = −2.19 × 10^–4^, FDR-adjusted *p* < 0.01). When normalized to baseline RA in substrate mixes, this shift corresponded to approximately 33% of the median baseline level (Table S4). In contrast, no significant directional shifts were detected for integrons, plasmid-associated MGEs, or other MGEs, whose median Δvalues remained close to zero and showed substantial variability among digesters (Table S4). When RA changes across all MGEs were aggregated, despite variability among plants, a statistically significant negative directional shift was observed (median Δ = −1.19 × 10^–4^, FDR-adjusted *p* < 0.01), corresponding to approximately 25% of the median baseline abundance in substrate mixes (Table S4). Similar to the consistency between AA and RA changes observed for ARGs during AD, RA changes of MGEs were generally consistent with corresponding trends in AA. Exceptions were observed in plants J and V, where a negative shift in RA coincided with an increase in AA. This discrepancy was likely driven by a substantial increase (6–10-fold) in absolute bacterial abundance in digestates relative to substrates in these plants. Consequently, despite a negative shift in RA, AA increased due to the overall proliferation of the microbial community.

Regarding postdigestion storage, no consistent directional shifts in RA of ARGs or MGEs were observed across the ten storage treatments (Figure S2). Median Δvalues for ARG classes and MGE categories remained close to zero, indicating that storage did not introduce systematic enrichment or depletion beyond the postdigestion state. Notably, in plant J, where liquid–solid separation of digestate was applied, the RA of ARGs and MGEs was consistently higher in the solid fraction than in the corresponding liquid fraction derived from the same digestate (Figure S2). Consistent with this pattern, AA of ARGs and MGEs was also substantially higher in the solid fraction than in the liquid fraction (Table 1). Together, these results indicate a preferential association of ARGs and MGEs with the solid fraction of digestate. However, comparable liquid–solid partitioning data were unavailable from the other selected biogas plants. Therefore, this observation should be interpreted as plant-specific rather than as a generalizable pattern.

### Effect of Operational and Physicochemical Parameters on ARG and MGE Dynamics during Anaerobic Digestion

Operational and physicochemical parameters were evaluated to explore their potential associations with changes in the RA of ARGs and MGEs during AD. For ARGs, samples exhibiting overall decreasing trends in RA tended to cluster in the direction of higher pH and NH_3_, as reflected by the positioning of samples such as K2_D, H1_D1, and H1_D2 along the direction of the pH and NH_3_ vectors (Figure 3A). This pattern suggests a potential association between elevated pH and NH_3_ levels and reductions in overall ARG RA. An exception was observed for S_SD, a secondary digester, which deviated from this general trend. For MGEs, most digesters displayed overall decreasing trends in RA, with only B_D and L_D showing increasing median RA values (Figure 2B). These samples were positioned opposite to the pH and NH_3_ vectors, consistent with the pattern observed for ARGs (Figure 3B).

**Figure 3:**
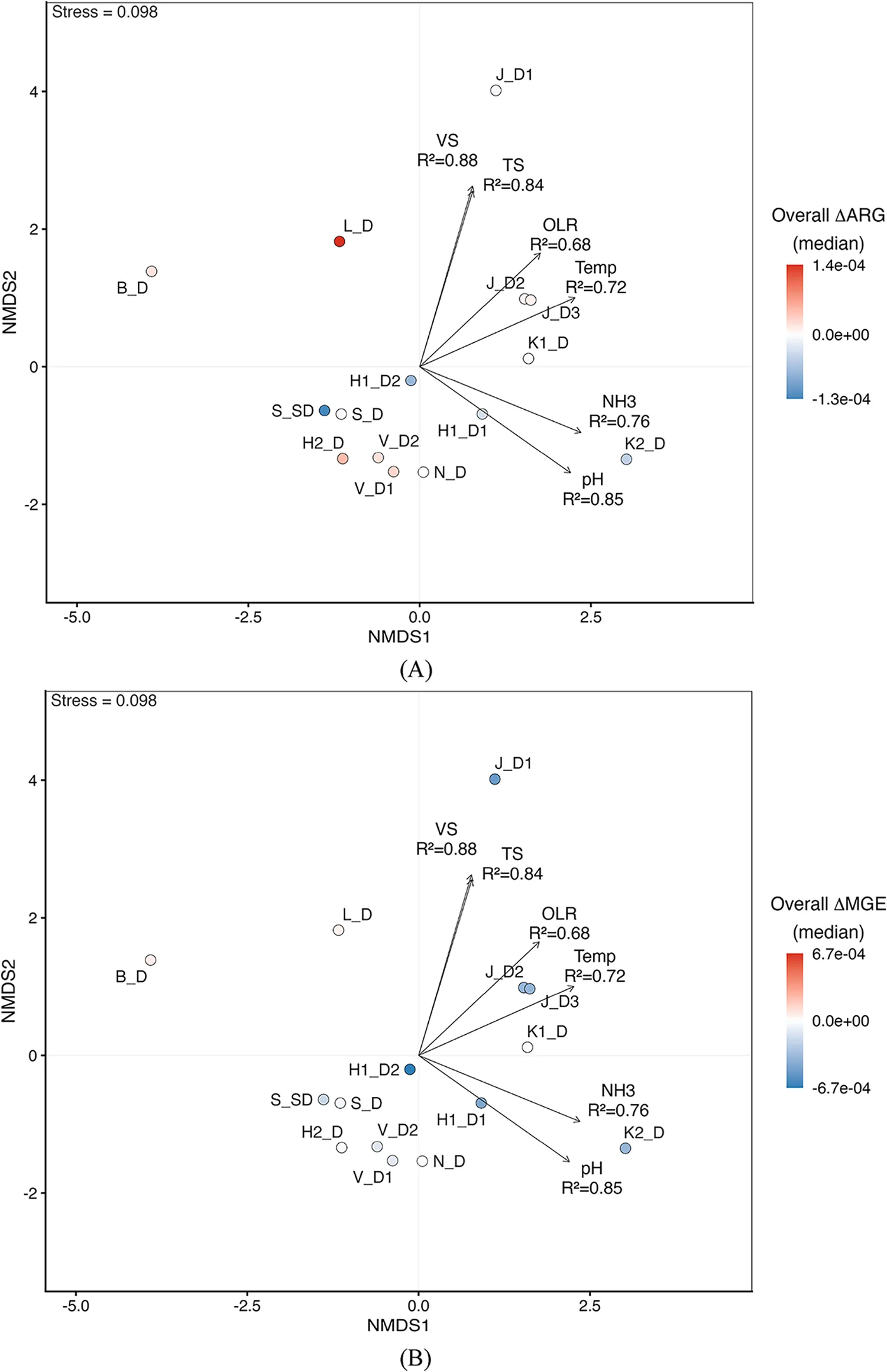
Nonmetric multidimensional scaling ordination of full-scale biogas digesters based on operational and physicochemical parameters. (A) Samples colored by the overall median change in antibiotic resistance genes (ΔARG). (B) Samples colored by the overall median change in mobile genetic elements (ΔMGE). Only variables showing significant correlations (*p* ≤ 0.05) are shown.

### Overview of the Putative Host-ARG-MGE Network Structure

A tripartite network integrating samples from substrates, digestates and postdigestion storages was constructed to capture associations among ARGs, MGEs and putative microbial hosts across full-scale biogas systems (Figure 4). All associations reported in the network analyses represent correlation-based coabundance associations and should therefore be interpreted as putative ecological associations rather than confirmed genetic associations or direct evidence of ARG carriage by specific microbial taxa. Based on robust positive correlations, the network comprised 67 ARG nodes, 30 MGE nodes, and 18 microbial phylum nodes, connected by a total of 204 edges. Distinct levels of connectivity were observed among the three network components, with MGEs exhibiting the highest number of associations, followed by ARGs, while microbial phyla showed the lowest connectivity. Among MGEs, several high-degree nodes were identified, including orf37-IS26 (*n* = 21), IS21-ISAs29 (*n* = 15), and ISCR1 (*n* = 12). Similarly, several ARGs displayed relatively high connectivity, such as *bla*_OXY_ (*n* = 12), *APH­(3́)-Ib* (*n* = 11), and *rmtB* (*n* = 7). In contrast, microbial phylum nodes exhibited substantially fewer associations, with Spirochaetota (*n* = 8), Abditibacteriota (*n* = 7), and Desulfobacterota (*n* = 3) representing the most connected phyla. Overall, the tripartite network revealed that ARG-MGE associations formed the dominant structural backbone, whereas microbial phyla occupied more peripheral positions, reflecting limited but specific associations. Because phylum-level resolution aggregates diverse genera with potentially distinct ARG and MGE profiles, these associations should be interpreted as broad community-level patterns rather than specific host-ARG-MGE genetic linkages. The genus-level networks described below were therefore used to further resolve putative bacterial taxa associated with ARGs and MGEs.

**Figure 4:**
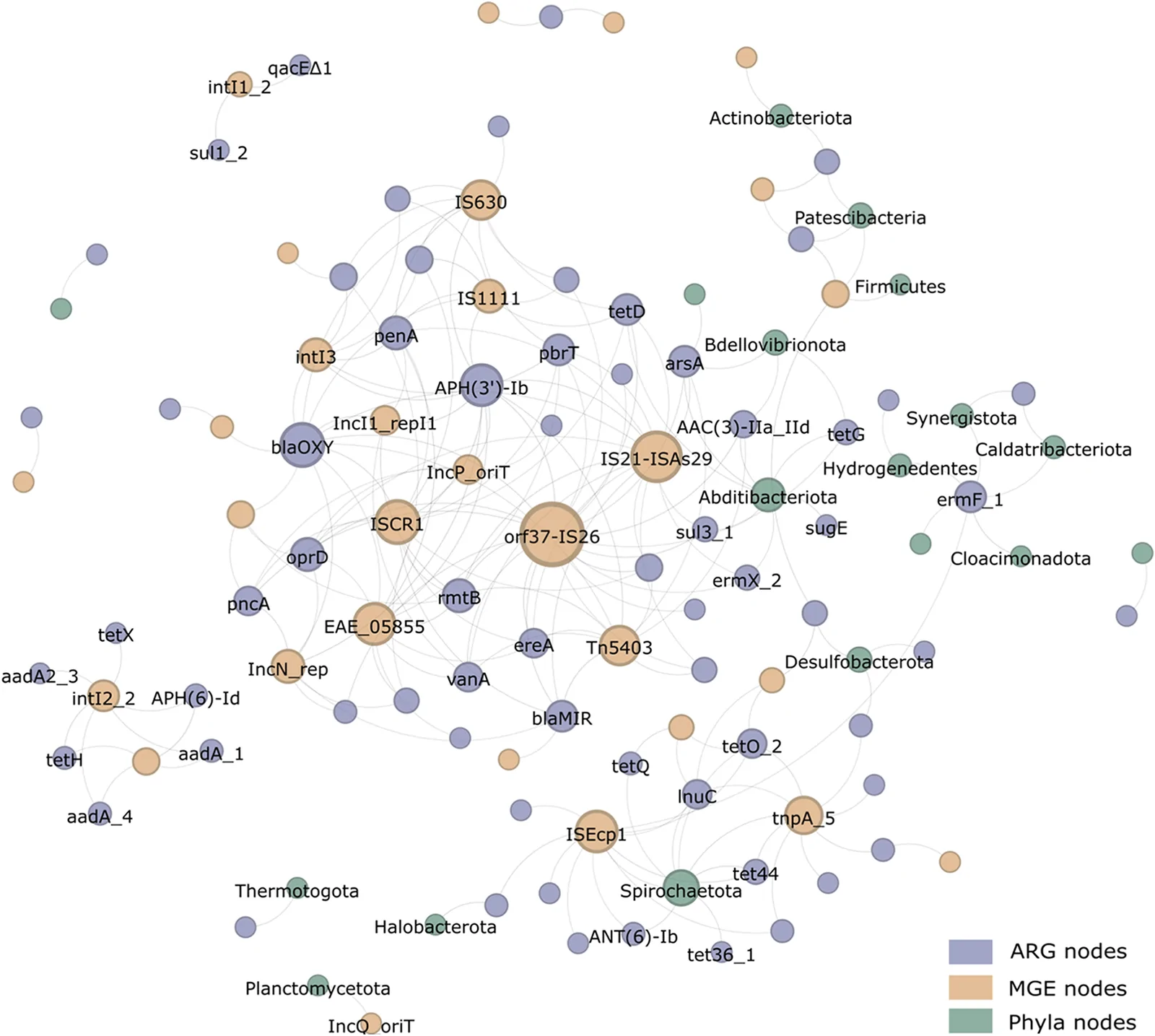
Tripartite network integrating antibiotic resistance genes (ARGs), mobile genetic elements (MGEs), and microbial phyla based on robust positive correlations (ρ > 0.6, FDR-adjusted *p* < 0.05). Node colors indicate ARGs, MGEs, and microbial phyla. Node size is proportional to node degree. Node labels are shown only for highly connected nodes or those discussed in the main text to improve visual clarity.

### Stage-Wise Dynamics of Putative Host-ARG-MGE Associations

To characterize the dynamics of associations among putative microbial hosts, ARGs, and MGEs across process stages, a global bipartite network was inferred from 42 samples, encompassing 17 substrates, 15 digestates, and 10 postdigestion storage samples. The resulting associations were subsequently evaluated within individual process stages (substrate, digestate, and postdigestion storage) to determine whether they were retained, lost, or newly supported across stages (Figure 5). Microbial associations with ARGs and MGEs were assessed at the genus level to provide sufficient taxonomic resolution.

**Figure 5:**
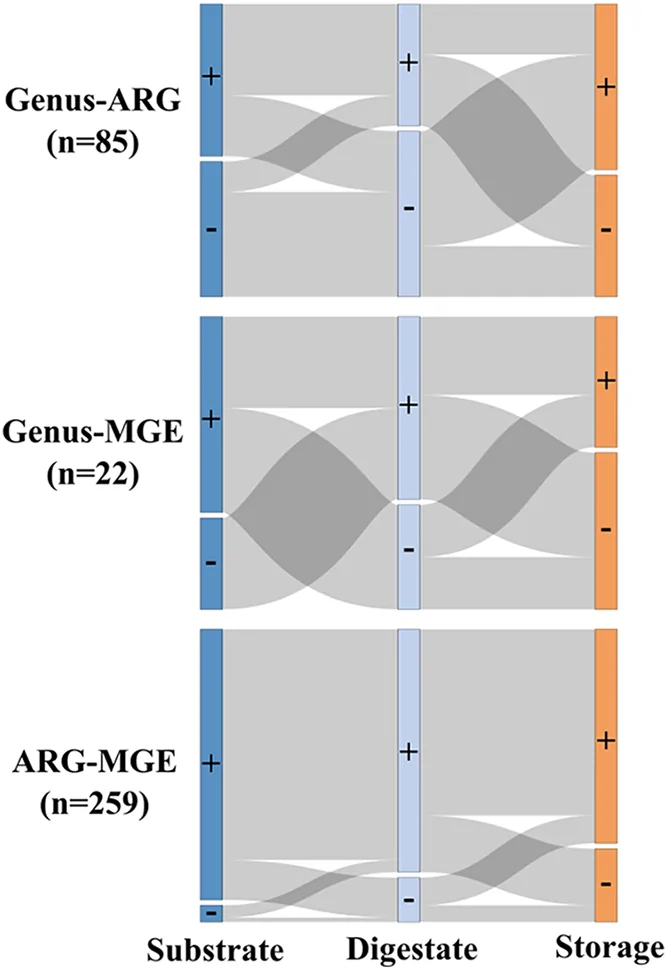
Bipartite Sankey diagrams illustrating stage-wise changes in Genus-ARG, Genus-MGE, and ARG-MGE coabundance associations across substrate, digestate, and storage stages. Bipartite associations were first inferred from the full data set using all samples, and the resulting association pool was subsequently evaluated for stage-specific support. Symbols “+” and “–” indicate whether an association was supported or not supported at a given stage. Flows represent the retention, loss, or emergence of associations between consecutive stages, reflecting stage-wise restructuring of association pools rather than persistence of individual links. Numbers in parentheses indicate the total number of associations included in each bipartite network.

For genus-ARG associations, stage-wise analysis revealed pronounced restructuring across the AD system (Figure 5). From substrate to digestate, a substantial proportion of identified genus-ARG associations was lost, accompanied by the emergence of a comparable number of new associations, reflecting marked shifts in ARG-associated taxa during the digestion process. This dynamic pattern became more pronounced during the transition from digestate to postdigestion storage, where newly formed genus-ARG associations exceeded retained ones and additional losses were observed. Together, these changes demonstrate continued reorganization of genus-ARG association patterns during postdigestion storage rather than stabilization after digestion. Topological analysis of the genus-ARG network showed a relatively even distribution of node degrees across bacterial genera, with no single genus acting as a dominant hub taxon (Table S5). Instead, ARG associations were distributed among multiple taxa, suggesting frequent reassortment of genus-ARG associations across successive process stages.

Compared with genus-ARG networks, genus-MGE associations were fewer in number and exhibited intermediate levels of stability across stages (Figure 5). From substrate to digestate, multiple genus-MGE associations were lost while new associations concurrently emerged. During the transition from digestate to storage, both retained and newly formed genus-MGE associations were observed, accompanied by similar losses compared to substrate-digestate transition. The continued appearance of new genus-MGE associations indicates ongoing redistribution of MGEs among microbial taxa beyond the digestion stage. Network topology analysis further showed that genus-MGE associations were markedly sparse, with most genera and MGEs exhibiting low connectivity and no pronounced hub structure (Table S5). This degree pattern reflects generally limited and transient taxon-MGE associations at the genus level.

In contrast, ARG-MGE associations exhibited a markedly different stage-wise pattern compared with taxon-associated networks (Figure 5). From substrate to digestate, the majority of ARG-MGE links were retained, with relatively few losses or newly formed associations, indicating strong conservation of resistance-mobility coupling during AD. This high degree of stability persisted during the transition from digestate to storage, where retained ARG-MGE associations dominated and only limited gains or losses were observed. Topological analysis of the ARG-MGE network indicated that connectivity was driven by a small number of highly connected MGEs (e.g., IS26-, ISEcp-, and IS21-associated elements; Table S5). These MGEs were repeatedly associated with multiple different ARGs across stages, whereas no individual ARG subtype exhibited comparable connectivity. Together, this indicates that ARG-MGE associations across stages are primarily structured by MGEs rather than by ARG-specific connectivity.

### Relative Abundance Patterns of ARG- and MGE-Associated Bacterial Genera

To place these network-associated bacterial genera in an abundance context, the RA of ARG- and MGE-associated genera was examined across samples and process stages (Figure 6). As shown in Figure 6, the majority of ARG- and MGE-associated bacterial genera occurred at appreciable RA, suggesting that these associations involve common taxa rather than being driven primarily by rare taxa. Overall, the number of ARG-associated genera was higher than that of MGE-associated genera. In total, these genera belonged to six bacterial phyla, with Firmicutes representing the dominant group, accounting for 18 out of 30 genera. The RA of ARG- and MGE-associated genera varied across samples and process stages (Figure 6). Although no consistent RA trends for these genera were observed across process stages, many genera exhibited relatively limited RA fluctuations, particularly those with high network degrees identified in the network analysis (Table S5). For example, the RA of *Acholeplasma* (*n* = 13) and *Syntrophomonas* *(n* = 8) remained comparatively stable across stages. In contrast, genera with lower network degrees showed more pronounced RA variability across process stages, including genera such as *Pantoea* and *W5*.

**Figure 6:**
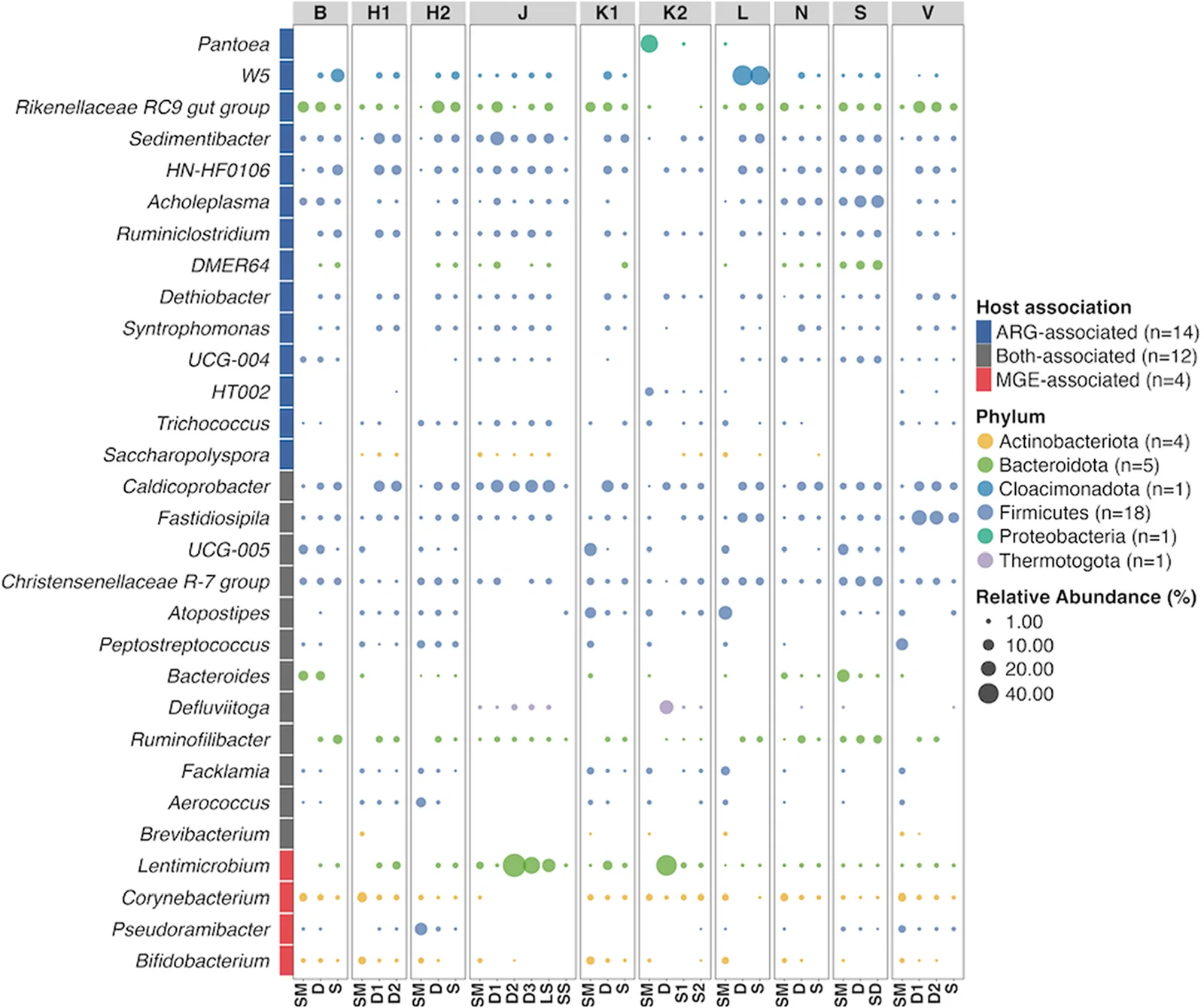
Distribution of bacterial genera associated with antibiotic resistance genes (ARGs) or mobile genetic elements (MGEs) across full-scale biogas digesters. For visual clarity, data points with relative abundance <0.1% are omitted from bubble display.

## Discussion

### Absolute Abundance Level of ARGs and MGEs in Agricultural Substrates under Low-Antibiotic-Use Conditions

Nordic countries have some of the strictest regulations on antibiotic use in agriculture worldwide, and Sweden was the first country to ban antibiotic growth promoters in animal feed as early as 1986.^30^ Under such stringent regulatory conditions, both AA levels and subtype-level variation patterns of ARGs and MGEs were similar among substrates from organic, conventional, and mixed conventional-organic systems (Figure S3). This suggests limited differences in antibiotic resistance loading associated with substrate origin in the present study.

Although direct quantitative comparison with existing studies remains challenging because of differences in substrate types, analytical platforms, target genes, and selection pressures, available studies still provide useful context. For example, a lab-scale wet and dry codigestion study used pig manure and food waste collected from a pig farm and university canteens in Galway, Ireland.^21^ In that study, the overall AA of ARGs and MGEs in the substrates was approximately 10^8^–10^9^ gene copies per gram wet mass, which is similar in order of magnitude to that observed here. Ireland represents a European antimicrobial-stewardship context in which antibiotic use in farm animals has been increasingly monitored and regulated, although data availability varies among livestock sectors.^31^ In contrast, another study reported that *ermB*, *tetM*, and *sul*2 were among the most frequently detected and abundant ARGs in 53 livestock manure samples from southeastern China.^32^ This represents a different antibiotic-use context, as food-animal production in China has historically involved comparably more intensive antibiotic use, although regulation has been strengthened in recent years.^33^ In that southeastern China study, the mean AA values exceeding 10^9^ gene copies per gram wet mass, whereas the corresponding mean AA values in the present study were generally within 10^7^–10^8^ gene copies per gram wet mass. These comparisons should be interpreted cautiously, but they suggest that differences in antibiotic-use context and associated selection pressures may contribute to variation in ARG loads in agricultural substrates entering AD systems. Taken together, the AA data generated in this study provide valuable baseline information on resistome-wide ARG and MGE loads in agricultural substrates entering full-scale AD systems under low-antibiotic-use conditions.

### Effects of Operational and Physicochemical Parameters on ARG and MGE AA and RA during Anaerobic Digestion

Across the investigated farm-scale AD systems, changes in AA and RA of ARGs and MGEs generally followed consistent directional patterns, with exceptions only in a limited number of plants where MGEs exhibited divergent trends between AA and RA. At the AD-system level, neither AA nor RA of ARGs showed a uniform directional trend across the 15 investigated digesters, with both increases and decreases observed depending on site-specific conditions (Figures 1 and[Fig fig2]). This heterogeneity indicates that AD cannot be assumed to universally reduce ARG AA or RA under real-world operational settings. Instead, the fate of ARGs appears to be highly context-dependent, shaped by plant-specific configurations and operating conditions as discussed below.

In the present study, samples exhibiting overall decreasing trends in ARG and MGE RA were generally positioned along gradients of higher pH and NH_3_ in the ordination analysis, indicating a potential association between these physicochemical conditions and the attenuation of resistance and mobility determinants (Figure 3). Notably, pH and NH_3_ concentrations are intrinsically linked in AD systems, as increases in pH are associated with changes in ammonia speciation toward a higher proportion of NH_3_. Previous studies have demonstrated that the influence of AD physicochemical parameters on ARG and MGE dynamics is predominantly mediated through changes in the microbial community, rather than through direct effects on genes themselves.^16,21^ In this context, NH_3_ has been reported to exert strong selective pressure on microbial community composition.^34^ Elevated NH_3_ concentrations have been shown to reduce microbial diversity during AD,^35,36^ and lower microbial diversity has, in turn, been associated with greater reductions in ARGs, likely reflecting a reduced availability of potential microbial hosts.^15,16^ More direct evidence for the role of NH_3_ in ARG attenuation has been provided by an experimental study by Zhang et al.^37^ who demonstrated that free ammonia pretreatment (420 mg NH_3_–N L^–1^ for 24 h) significantly enhanced the reduction in AA of multiple ARGs, including *aac­(6′)-Ib-cr*, *bla*_TEM_, *sul*2, *tetA*, *tetB*, and *tetX*, during subsequent AD. Consistent with this finding, our previous study also observed enhanced ARG reduction under higher NH_3_ levels in agricultural biogas plants treating plant-based substrates.^9^

However, elevated pH and NH_3_ levels alone were not determinative of ARG and MGE dynamics. In the present study, for example, digesters H1_D2 and V_D2 exhibited divergent fates of ARGs and MGEs, with H1_D2 showing an overall reducing pattern and V_D2 exhibiting an enriching pattern (Table 1), despite comparable pH and NH_3_ concentrations (Table S2). This indicates that combined pH-NH_3_ conditions were insufficient to fully explain resistance gene dynamics. This divergence of gene fates likely reflects the influence of additional operational and physicochemical parameters acting in concert with pH and NH_3_. Among these factors, temperature is one of the most extensively studied and widely regarded as a critical parameter shaping microbial community structure in AD systems.^10,38^ Numerous studies have reported that thermophilic AD generally achieves greater reductions in ARGs than mesophilic operation.^11,16,17^ For instance, Diehl and LaPara^39^ demonstrated that increasing digestion temperature from 22 to 55 °C significantly enhanced the removal of tetracycline resistance genes and the class I integron integrase gene *intI*1, highlighting the sensitivity of both ARGs and integron-associated elements to thermal conditions. More recently, Zou et al.^14^ showed that thermophilic digestion (55 °C) promoted the reduction of extracellular ARGs and *intI*1 compared with mesophilic operation (35 °C), suggesting that elevated temperature may facilitate the degradation of both intracellular and extracellular genetic material. In this context, the contrasting ARG and MGE trajectories observed in digesters H1_D2 and V_D2 may be partly attributable to differences in operating temperature, with H1_D2 operating at 41 °C compared with 38 °C in V_D2. In addition to temperature, other physicochemical parameters may also have contributed to the divergent ARG and MGE fates observed in H1_D2 and V_D2, including differences in OLR, VS, and TS, as suggested by the ordination analysis (Figure 3). However, existing studies provide limited evidence for consistent or direct associations between these parameters and ARG or MGE reduction under typical wet AD conditions. Notably, enhanced ARG and MGE reduction associated with higher TS has primarily been reported for high-solids or dry AD systems (TS > 15%), which have been shown to outperform wet-type systems (TS < 8%) in terms of ARG attenuation.^20,21^ In the present study, TS values in both H1_D2 and V_D2 remained below 8%, suggesting that differences in TS within this low range are unlikely to represent a dominant driver of the contrasting resistance gene dynamics observed between these digesters, despite the relatively higher TS in H1_D2. Moreover, the microbial community structure may be influenced by the combined effects of these parameters. H1_D2 exhibited weaker associations with dominant microbial phyla than V_D2 (Figure S4), which may be linked to the observed reduction pattern in H1_D2.

Collectively, among the operational and physicochemical parameters examined across the 15 digesters, elevated pH and NH_3_ were the parameters most consistently associated with effective ARG and MGE reduction, likely through their influence on microbial community structure. Nevertheless, the combined pH-NH_3_ condition alone was not solely determinative of ARG and MGE dynamics, and the observed variability among digesters indicates that multiple operational and physicochemical parameters act in concert to shape ARG and MGE fate during full-scale AD. A more definitive assessment of the effects of individual operational parameters, such as temperature, pH, NH_3_, HRT, and OLR, will require a larger plant-level data set with greater statistical power and more balanced operational contrasts.

It is noteworthy that transposon-related MGEs exhibited a statistically significant negative directional shift during AD, whereas other MGE categories did not. This is consistent with a previous study showing that different MGE categories can respond differently during digestion, with transposase genes decreasing and integrase genes increasing.^40^ In our study, this decrease may reflect the reduction or replacement of bacterial populations carrying transposon-related markers. Such carrier turnover could occur during the transition from substrate communities to AD-adapted anaerobic communities. In contrast, plasmid-associated markers and integrons may have greater opportunities to be maintained across different mobile platforms or host backgrounds, as plasmids can persist through replication and, in some cases, horizontal transfer, while integrons are frequently embedded within plasmids or transposons.^41,42^ This may partly explain why transposon-related MGEs showed a consistent class-level decline, whereas plasmid-associated MGEs and integrons did not show significant directional shifts under the investigated conditions. This interpretation remains putative, and metagenomic or long-read sequencing would be required for further validation.

### Effect of System Configurations on ARG and MGE AA and RA

Beyond operational and physicochemical parameters, process configuration has also been shown to play an important role in shaping the dynamics of ARGs and MGEs during AD. Two-phase digestion systems have repeatedly been reported to achieve greater reductions in ARGs and *intI*1 than single-phase systems, with the acidogenic phase often identified as a critical window for resistance attenuation, followed by more variable responses during the methanogenic stage.^19,43^ Evidence from full-scale systems further supports this pattern. For example, Jang et al.^18^ reported significant reductions of ARGs and *intI*1 in two-phase thermophilic digesters, with several resistance genes becoming undetectable in the final effluent. It should be noted that the secondary digester examined in the present study does not represent a classical acidogenic-methanogenic two-phase configuration. In plant S, the primary digester operated at near-neutral pH (7.67) with a relatively long HRT (29 d), during which the main AD processes were already completed. The secondary digester (pH 7.48; HRT 4 d) therefore functioned primarily as a postdigestion polishing unit by extending the overall residence time, rather than introducing a distinct metabolic phase. Despite the different configuration, the two-stage system in plant S still exhibited pronounced reductions in ARGs and MGEs. One possible explanation is the comparably more oligotrophic conditions in the secondary reactor, which has been shown to be associated with reduced microbial diversity and enhanced ARG removal.^44,45^ This interpretation is supported by the comparison of microbial community structures between the primary and secondary digesters (Figure S5), where both the RA and diversity of genera appeared to be lower in the secondary digester compared with the primary digester. In addition, prolonged residence time may promote antibiotic biodegradation,^46^ potentially resulting in lower residual antibiotic concentrations in the secondary digester and, consequently, reduced selective pressure for antibiotic resistance. Overall, although the two-stage configuration in plant S differs from a classical acidogenic-methanogenic system, it nevertheless exhibited an effective reduction of ARGs and MGEs. This finding may suggest that extending residence time through multistage configurations may also lead to enhanced ARG and MGE reduction, similar to outcomes reported for classical two-phase digestion. In this context, two-stage operation can represent a potentially effective approach for reducing ARGs and MGEs.

In full-scale biogas systems, postdigestion storage is routinely implemented to balance temporal mismatches between digestate production and agricultural application, to stabilize digestate properties, and to facilitate subsequent handling practices such as solid–liquid separation.^47,48^ Beyond these operational functions, postdigestion storage has also been recognized as a stage at which further changes in microbial activity and associated genetic elements may occur.^49,50^ In the present study, postdigestion storage exhibited heterogeneous effects on ARG and MGE abundances, with both increases and decreases observed across storage treatments in terms of both AA (Table 1) and RA (Figure S2). Such variability is consistent with previous studies, which have shown that ARG dynamics during storage are highly gene- and subtype-specific rather than uniform. For example, Zhang et al.^49^ reported that during 30 days of digestate storage, total ARG RA decreased overall, while several ARG subtypes, including *tetM*, *tetX*, *tetQ*, *tetS*, *ermF*, and *sul*2, persisted or increased. Similarly, Pu et al.^50^ observed pronounced enrichment of ARGs in RA during 28 days of aerobic storage of biogas residues. Together, these findings indicate that individual resistance genes may exhibit divergent responses during storage, even when overall RA trends appear limited or inconsistent.

Beyond gene-level heterogeneity, postdigestion storage has also been reported to influence the partitioning of ARGs between solid and liquid fractions. Zhang et al.^48^ showed that following AD of dairy manure, ARG AA decreased in the liquid fraction but was concurrently enriched in the solid fraction, with higher TS content associated with increased ARG concentrations in solids. This pattern is consistent with our observations from solid–liquid separated storage in plant J, where both AA and RA of ARGs were consistently higher in the solid fraction than in the corresponding liquid fraction (Table 1 and Figure S2). The higher abundance in the solid fraction may partly be explained by the higher concentration of DNA-bearing material in this fraction, where microbial cells, biofilms, flocs, and extracellular DNA can be associated with suspended solids and organic particles.^51,52^ Collectively, these results suggest that postdigestion storage represents a complex and heterogeneous stage with respect to ARG and MGE dynamics, characterized by gene-specific responses and phase-dependent distributions rather than uniform enrichment or reduction.

### Network Analysis of Putative Host-ARG-MGE Associations

Reliable inference of microbiome association networks critically depends on adequate sample size and appropriate statistical control, particularly in high-dimensional data sets involving microbial taxa, ARGs and MGEs. Recent studies have cautioned that networks inferred from fewer than 20 samples per group are prone to instability and spurious associations.^53,54^ This study used the global network based on all 42 samples and then evaluated stage-wise support of these associations within individual process stages. This approach, together with CLR transformation and FDR-adjusted *p*-values, provided a stable framework for interpreting putative host-ARG-MGE associations across the AD system.

The conventional tripartite network highlighted a pronounced structural imbalance among putative host taxa, ARGs, and MGEs (Figure 4). ARG-MGE associations formed the dominant backbone of the network, whereas taxa putatively associated with ARGs or MGEs occupied more peripheral positions with comparatively fewer connections. This pattern is consistent with several previous network-based AD studies reporting limited putative taxon-ARG associations.^11,19,55^ In contrast, some studies have reported more extensive associations between microbial phyla and ARGs or MGEs.^21,25^ Such discrepancies in the number of associations involving microbial taxa, ARGs and MGEs, may be attributable to methodological differences, including limited sample sizes (e.g., *n* = 6)^21^ or the use of less stringent correlation thresholds,^25^ both of which can increase the likelihood of detecting spurious associations.

Network analyses in previous AD studies are commonly derived from aggregated samples and are often used to infer putative microbial hosts or taxa associated with ARGs or MGEs, thereby linking changes in ARG and MGE abundances to shifts in microbial community composition along the AD process. For example, enhanced ARG reduction has been attributed to the elimination of putative host bacteria during digestion.^56^ However, this interpretation is challenged by the dynamic putative host-ARG-MGE associations observed in the present study. Although ARG-MGE associations remained comparatively stable across process stages, the putative microbial hosts associated with ARGs and MGEs shifted substantially from substrate to digestate and further to postdigestion storage. Consequently, association patterns inferred from aggregated samples represent static snapshots that may not fully capture stage-specific dynamics. The present study provides a more resolved, stage-specific perspective, highlighting the dynamic nature of associations across the AD process.

An interesting observation emerged when putative host-ARG-MGE associations were examined at different taxonomic resolutions. Specifically, the identity of the most strongly associated phyla differed depending on whether microbial taxa were resolved at the genus or phylum level. At the genus level, the majority of highly associated taxa belonged to Firmicutes, followed by Actinobacteriota, which is consistent with findings reported in many previous studies (Figure 6).^25,55,56^ In contrast, when associations were analyzed directly at the phylum level, Spirochaetota and Abditibacteriota emerged as the most highly associated phyla. In the present study, Spirochaetota exhibited associations with multiple ARGs, including *tet*44, *tet*36, *tetQ*, *lnuC*, and *ANT­(6)-Ib*, as well as with MGEs such as ISEcp1 and *tnpA* (Figure 4). *Treponema*, a representative belonging to Spirochaetota was also observed at relatively high abundance (Figure S5). Consistent with previous reports, *Treponema* has frequently been associated with ARGs, particularly tetracycline resistance genes, in AD and related environments.^45,48,57^ By contrast, phylum Abditibacteriota has rarely been reported in AD systems, likely due to its generally low relative abundance (<1%). Nevertheless, it exhibited associations with multiple ARGs spanning different antibiotic classes, including *tetD*, *tetG*, *sul*3, *ermX*, *sugE*, *arsA*, and *AAC­(3)-IIa_IId*, although no associations with MGEs were detected (Figure 4). Abditibacteriota has previously been reported to display high phenotypic resistance to a broad range of antibiotics.^58^ Overall, the inconsistency in top associated phyla across taxonomic levels likely reflects the fact that phylum-level taxa encompass diverse genera with distinct resistance-related roles. As a result, genus-specific putative host-ARG-MGE associations may be masked or diluted when analyses are conducted at broader taxonomic resolutions.

To our knowledge, this study represents the first application of a stage-resolved network framework to investigate putative host-ARG-MGE associations across the full-scale AD and postdigestion storage process, moving beyond static correlation analyses based on aggregated samples. Using this approach, we show that although ARG-MGE associations remain largely conserved across process stages, the microbial taxa associated with these resistance determinants undergo continuous reshuffling throughout the AD system. These findings suggest that ARG persistence in full-scale biogas plants is driven less by stable associations with specific taxa and more by the redistribution of ARG-MGE association patterns among different microbial taxa, i.e., AD functions primarily as a process of taxonomic association reshuffling rather than complete resistance elimination. This framework provides a potential mechanistic explanation for why abundance-based assessments alone often fail to resolve the apparent stability of the resistome across AD and postdigestion storage. Consistent with this interpretation, the AA of ARGs and MGEs in this study remained within a similar order of magnitude (10^8^–10^10^ gene copies per gram wet mass) from substrates through digestates and subsequent storage, despite pronounced changes in microbial community composition and putative host associations.

### Environmental Implications

This study demonstrates that digestate storage should not be regarded as a passive holding phase prior to land application. Instead, storage represents a biologically active stage during which ARGs and MGEs may continue to be redistributed within microbial communities, reshaping host-gene associations beyond the digestion end point. Such dynamics imply that resistance determinants introduced into agricultural soils may reflect ongoing postdigestion processes rather than solely conditions at digester exit. At the same time, these effects should be interpreted in the context of the substantial initial ARG and MGE loads present in raw substrates. Against this large baseline, the moderate reductions or enrichments observed during AD and storage are unlikely to substantially alter AA inputs to the environment. Therefore, digestate remains an environmentally preferable alternative to untreated substrates such as raw manure, owing to its improved nutrient bioavailability, higher nutrient use efficiency, and comparable ARG and MGE levels relative to raw substrates. Overall, AD and subsequent storage do not eliminate resistance determinants but modify their ecological context prior to land application. Environmental risk assessments should therefore move beyond abundance-based metrics alone and incorporate postdigestion dynamics alongside agronomic benefits when evaluating digestate use in sustainable agricultural systems.

## Supplementary Material



## Notes

Additional details on physicochemical analyses; DNA extraction, qPCR assays, and 16S rRNA gene sequencing; sampling schemes and operational characteristics of full-scale biogas plants; classification of ARGs and MGEs; calculation of ARG and MGE absolute abundances; abundance distributions and changes across substrates, digestates, and storage; bacterial community composition; and coabundance network characteristics (PDF)
