# Supplementary material for: Stage-Resolved Dynamics of Antibiotic Resistance Genes and Mobile Genetic Elements in Full-Scale Anaerobic Digestion Systems
Source: Environ Sci Technol. 2026 Oct 31;60(44):31930–43. doi: 10.1021/acs.est.6c04399 (PMC13757947; doi:10.1021/acs.est.6c04399)
Supplement: Supplementary file 1 [file es6c04399_si_001.pdf]

Stage-Resolved Dynamics of Antibiotic Resistance Genes and Mobile Genetic Elements in Full-scale Anaerobic Digestion Systems

He Sun, †, Sara B. Nilsson‡, Anna Schnürer*, †*

†Department of Molecular Sciences, Swedish University of Agricultural Sciences, Uppsala, Sweden

‡Hushållningssällskapet, Halland, Sweden

Table S1. Overview of samples collected from ten full-scale biogas plants in Sweden, including substrates and sampling points across the anaerobic digestion process.

No.	Biogas plants	Substrates							Digestates							
		Cow manure	Pig manure	Chicken manure	Deep bedding	Food waste	Slaughter-house waste	Plant materials	Primary digester1	Primary digester2	Primary digester3	Secondary digester	Liquid separation	Solid separation	Storage1	Storage2
1	H1	X		X	X			X	X	X						
2	H2	X							X						X	
3	L	O	O					X	X						X	
4	V	O	O	O		O	O		X	X					X	
5	K2	O	O		△	△		X	X						X	X
6	B	O	O						X						X	
7	J							X	X	X	X		X	X		
8	K1	X							X						X	
9	S	O			O				X			X				
10	N		O		O				X						X	

“X” indicates individual samples collected from a single substrate or process unit. “O” and “△” indicate samples obtained from premixed materials prior to sampling (i.e., composite samples representing multiple substrate inputs). Plant numbers highlighted in green (plants 1–3) represent organic farming systems, those in yellow (plants 4–7) represent mixed conventional–organic systems, and those in red (plants 8–10) represent conventional farming systems.

Table S2. Operational parameters and sample characteristics of the ten full-scale biogas plants included in this study.

Biogas plants	Samples	OLR[a], kg (VS[b])•m^{-3}•d^{-1}	TS[c], %	VS, % of TS	pH	NH_4^+-N, mg•L^{-1}	NH_3[d], mg•L^{-1}	VFA[e], g•L^{-1}	HRT[f], d	Temp, ℃
H1	Cow manure	2.95	11.21±1.26	58.26±5.08	7.01	1142	-	7.54	-	-
	Deep bedding		23.72±2.45	63.98±2.23	-	-	-	-	-	-
	Oat water		15.70±1.62	69.66±2.04	4.44	115	-	10.12	-	-
	Chicken manure		43.20±0.48	76.44±1.47	-	-	-	-	-	-
	Digestate 1		6.01±0.43	41.80±1.14	7.85	2800	294	0.95	28	41
	Digestate 2		5.46±0.21	38.98±1.14	7.45	2403	107	0.27	28	41
H2	Substrate mix	1.38	5.93±1.27	42.02±6.46	6.58	1560	-	7.18	-	-
	Digestate		4.29±0.83	33.42±4.07	7.39	1998	69	0.85	18	39
	Storage		2.86±0.17	23.25±2.00	7.32	1449	-	1.90	na[h]	na[h]
L	Solid substrate	1.82	77.04±0.55	85.69±2.09	-	-	-	-	-	-
	Liquid substrate		na[g]	na[g]	7.72	1611	-	4.28	-	-
	Digestate		9.23±0.17	51.46±1.89	7.46	1983	71	0.14	55	37
	Storage		6.43±0.18	44.52±2.04	7.45	2565	-	0.11	na[h]	na[h]
V	Substrate mix	1.00	7.75±0.10	50.15±1.24	6.57	2061	-	11.49	-	-
	Digestate 1		5.15±1.65	30.70±3.60	7.69	3060	186	0.45	30	38
	Digestate 2		4.45±0.35	33.36±2.42	7.52	3090	130	0.67	30	38
	Storage		1.92±0.10	16.17±2.37	7.74	2400	-	0.25	na[h]	na[h]
K2	liquid manure	4.45	7.59±0.09	52.39±0.29	7.29	1704	-	5.50	-	-
	Solid manure		35.81±0.30	74.78±1.12	-	-	-	-	-	-
	Agnar		85.03±0.06	80.74±0.65	-	-	-	-	-	-
	Digestate		5.55±0.56	38.03±3.30	8.01	2127	509	0.18	32	51
	Storage 1		5.04±0.49	37.16±2.63	7.66	2178	-	0.95	30	na[h]
	Storage 2		4.18±0.09	31.33±2.18	7.86	2307	-	1	30	na[h]
B	Substrate mix	3.11	9.16±0.10	58.18±1.57	6.55	2043	-	10.86	-	-
	Digestate		7.33±0.07	49.87±0.83	6.87	2004	21	7.10	30	39
	Storage		4.57±0.02	32.81±0.87	7.70	2187	-	0.49	21	na[h]
J	Liquid substrate	5.29	7.78±0.01	61.45±2.68	3.58	128	-	25.49	-	-
	Solid substrate		38.79±0.89	78.28±1.32	-	-	-	-	-	-
	Digestate 1		32.69±0.46	68.83±2.52	7.30	1563	72	0.75	36	47
	Digestate 2		11.93±0.12	63.35±1.54	7.66	2412	240	0.32	36	47
	Digestate 3		13.06±0.07	62.09±1.26	7.66	2532	252	0.44	36	47
	Liquid storage		11.91±0.11	61.12±1.91	7.67	2004	-	0.43	0	na[h]
	Solid storage		6.22±0.02	44.53±3.10	-	-	-	-	10	na[h]
K1	Substrate mix	6.68	9.40±0.16	52.27±3.04	7.14	1812	-	0.22	-	-
	Digestate		7.06±0.15	46.37±1.07	7.82	2148	225	0.57	17	42
	Storage		3.65±0.31	31.27±0.70	8.15	1473	-	0.33	70	na[h]

S	Substrate mix	0.60	4.62±0.05	38.10±1.32	6.98	1458	-	5.35	-	-
	Digestate		4.01±0.02	36.83±0.82	7.67	1776	89	0.08	29	35
	Secondary digestate		3.94±0.01	31.59±0.67	7.48	1788	59	0.11	33	35
N	Substrate mix	0.78	5.43±0.17	40.15±1.93	6.99	1935	-	7.99	-	-
	Digestate		3.14±0.02	27.79±1.24	7.80	1788	160	0.13	28	40
	Storage		1.56±0.06	15.27±1.15	7.66	1863	-	0.16	21	na[h]

[a]Organic loading rate. [b]Volatile solids. [c]Total solids. [d]Free ammonia concentration calculated according to Calli et al. (2005). [e]Volatile fatty acids. [f]Hydraulic retention time. For digestate samples, this represents the hydraulic retention time of the biogas plant; for storage samples, it represents the storage duration prior to use as biofertiliser. [g]Not analysed due to insufficient sample quantity. [h]Information unavailable.

Table S3. Classification of antibiotic resistance gene (ARG) subtype and mobile genetic element (MGE) analyzed in this study.

No.	ARG class	ARG subtype	ARG class	ARG subtype	MGE category	MGE element
1	MLSB	ermF_1	Tetracycline	tetW	Integron	intI1_1
2	MLSB	ermF	Tetracycline	tetM	Integron	intI3
3	MLSB	lnuB	Tetracycline	tetQ	Integron	intI1_2
4	MLSB	ermX_2	Tetracycline	tet(44)	Integron	intI2
5	MLSB	mel_1	Tetracycline	tetD	Plasmid-associated	IncQ_oriT
6	MLSB	mphA	Tetracycline	tet(36)_1	Plasmid-associated	IncI1_repI1
7	MLSB	ermB_2	Tetracycline	tetG	Plasmid-associated	IncP_oriT
8	MLSB	ermB_1	Tetracycline	tetX	Plasmid-associated	IncW_trwAB
9	MLSB	ereA	Tetracycline	tetO_2	Plasmid-associated	IncN_rep
10	MLSB	ermB_3	Tetracycline	tetT	Plasmid-associated	trfA
11	MLSB	lnuC	Tetracycline	tetL_2	Plasmid-associated	IncF_FIC
12	MLSB	ermE	Tetracycline	tetH	Plasmid-associated	IncHI2-smr0018
13	MLSB	pncA	Tetracycline	tetR	Plasmid-associated	pAMBL
14	MLSB	mel	Tetracycline	tetB(P)_1	Plasmid-associated	pAKD1
15	MLSB	lnuF	Tetracycline	tet(32)	Plasmid-associated	IncN_korA
16	MLSB	ermA	Tetracycline	tetA_2	Plasmid-associated	IncN_oriT
17	MLSB	ermC_2	Tetracycline	tetS	Transposon-related	IS6/257
18	MLSB	lnuA_1	Tetracycline	tet(39)	Transposon-related	tnpA_6
19	MLSB	erm(31)	Tetracycline	tetA/B_1	Transposon-related	tnpA_7
20	MLSB	vatB	Tetracycline	tetR_1	Transposon-related	ISEcp1
21	MLSB	ermO	Tetracycline	tetJ	Transposon-related	tnpA_5
22	MLSB	ermY	Tetracycline	tetK	Transposon-related	IS1111
23	MLSB	mefB	Tetracycline	tetE	Transposon-related	orf37-IS26
24	MLSB	msrE	Tetracycline	tetA(P)_1	Transposon-related	Tn5403
25	MLSB	erm(35)	Tetracycline	tetC_2	Transposon-related	IS256
26	MLSB	erm(42)	Tetracycline	tet(38)	Transposon-related	IS1247_1
27	MLSB	vatA	Vancomycin	vanA	Transposon-related	IS613
28	MLSB	oleC	Vancomycin	vanTC_2	Transposon-related	tnpA_1
29	MLSB	ermX_1	Vancomycin	vanYD_1	Transposon-related	IS6100
30	MLSB	msrA_1	Vancomycin	vanHB	Transposon-related	IS630
31	MLSB	lmrA_1	Vancomycin	vanB_1	Transposon-related	IS26_1
32	MLSB	erm(36)	Vancomycin	vanC_5	Transposon-related	tnpA_2
33	MLSB	vatE_2	Vancomycin	vanTE	Transposon-related	tnpA_3
34	MLSB	ermD_2	Vancomycin	vanYB	Transposon-related	IS1133
35	MLSB	vgaB_1	Vancomycin	vanRB	Transposon-related	IS1247_2
36	MLSB	ermH	Vancomycin	vanSC_2	Transposon-related	ISCR1
37	MLSB	ermT_1	Vancomycin	vanHD	Transposon-related	IS21-ISAs29
38	MLSB	mphB	Vancomycin	vanRA_1	Transposon-related	Tp614
39	MLSB	erm(34)	Vancomycin	vanTG	Transposon-related	ISEfm1
40	MLSB	ermD	Vancomycin	vanC_2	Transposon-related	Tn3

41	MLSB	ermA_2	Vancomycin	vanG	Transposon-related	IS3
42	MLSB	vgaA_1	Vancomycin	vanRC	Transposon-related	ISAba3
43	MLSB	msrC_1	Vancomycin	vanRC_2	Transposon-related	Tn5
44	MLSB	lsaC	Vancomycin	vanSB	Transposon-related	IS5/IS1182
45	MLSB	ermD_1	Vancomycin	vanXB	Transposon-related	IS200_2
46	MLSB	vga(A)LC_1	Vancomycin	vanWB	Transposon-related	tnpA_4
47	MLSB	mel_3	Vancomycin	vanRD	Transposon-related	ISPps
48	Quinolone	qepA	Vancomycin	vanD	Transposon-related	IS200_1
49	Quinolone	qnrB_3	Vancomycin	vanSA	Other-MGE	trbC
50	Quinolone	norA	Vancomycin	vanXA	Other-MGE	EAE_05855
51	Quinolone	qnrB_2	Trimethoprim	dfrA17_1	Other-MGE	traN
52	Quinolone	qnrS2	Trimethoprim	dfra17	Other-MGE	cro
53	Quinolone	qnrD	Trimethoprim	dfrA1_1		
54	Quinolone	qnrB	Trimethoprim	dfrA27		
55	Quinolone	qnrVC1_VC3_VC6	Trimethoprim	dfrA25		
56	Quinolone	qnrVC_2	Trimethoprim	dfrB		
57	Quinolone	qnrA	Trimethoprim	dfrA15		
58	Quinolone	qnrS_1	Trimethoprim	dfrA22		
59	Beta Lactam	penA	Trimethoprim	dfrA12_1		
60	Beta Lactam	blaOXY	Trimethoprim	dfrG		
61	Beta Lactam	blaSFO	Trimethoprim	dfrK		
62	Beta Lactam	blaGOB	Trimethoprim	dfrA8		
63	Beta Lactam	cfxA	Trimethoprim	dfrA12		
64	Beta Lactam	blaOXY-1	Trimethoprim	dfrA10		
65	Beta Lactam	blaMIR	Trimethoprim	dfrC		
66	Beta Lactam	blaACT	Trimethoprim	dfrA19		
67	Beta Lactam	blaFOX	Trimethoprim	dfrAB4		
68	Beta Lactam	blaCTX-M	Aminoglycoside	aadA7		
69	Beta Lactam	blaSHV_4	Aminoglycoside	aadA_4		
70	Beta Lactam	blaOXA-48	Aminoglycoside	aadA_1		
71	Beta Lactam	blaNDM	Aminoglycoside	ANT(6)-Ia		
72	Beta Lactam	cphA_1	Aminoglycoside	aadA2_3		
73	Beta Lactam	blaDHA	Aminoglycoside	APH(6)-Ic		
74	Beta Lactam	blaOCH	Aminoglycoside	APH(7")-Ib		
75	Beta Lactam	blaCMY_2	Aminoglycoside	AAC(3)-IIa_IId		
76	Beta Lactam	blaCARB	Aminoglycoside	APH(3')-Ib		
77	Beta Lactam	blaACC	Aminoglycoside	APH(6)-Id		
78	Beta Lactam	blaROB	Aminoglycoside	ANT(6)-Ib		
79	Beta Lactam	ccrA	Aminoglycoside	AAC(3)-III		
80	Beta Lactam	cfiA	Aminoglycoside	APH(9)-Ib		
81	Beta Lactam	pbp5	Aminoglycoside	APH(6)-Ia		
82	Beta Lactam	blaOXA-51	Aminoglycoside	aadA16		
83	Beta Lactam	blaCTX-M-5	Aminoglycoside	ANT(6)-Ia_1		

84	Beta Lactam	blaPER	Aminoglycoside	AAC(3)-Iva
85	Beta Lactam	blaMOX/blaCMY	Aminoglycoside	ANT(2")-Ia
86	Beta Lactam	pbp	Aminoglycoside	aadA6
87	Beta Lactam	blaL1	Aminoglycoside	rmtB
88	Beta Lactam	imiR	Aminoglycoside	APH(3')-Ia
89	Beta Lactam	blaPDC	Aminoglycoside	AAC(3)-Ib
90	Beta Lactam	blaVIM	Aminoglycoside	AAC(6')-Ir
91	Beta Lactam	blaSME	Aminoglycoside	aadA2_1
92	Beta Lactam	blaTEM	Aminoglycoside	APH(3')-IIIa_1
93	Beta Lactam	blaADC	Aminoglycoside	APH(3')-Ia
94	Beta Lactam	blaBEL	Aminoglycoside	AAC(3)-Xa
95	Beta Lactam	blaKPC	Aminoglycoside	AAC(3)-IVa_1
96	Beta Lactam	blaHERA	Aminoglycoside	AAC(6')-IIc
97	Beta Lactam	blaACC-1	Aminoglycoside	ANT(4')-Ib
98	Beta Lactam	blaSST	Aminoglycoside	apmA
99	Beta Lactam	blaIMI	Aminoglycoside	aadA10
100	Beta Lactam	blaCARB_1	Aminoglycoside	aadA9_1
101	Beta Lactam	cepA	Aminoglycoside	AAC(6')-Iz
102	Beta Lactam	blaLEN	Aminoglycoside	APH(7")-Ia
103	Beta Lactam	mecA	Aminoglycoside	AAC(3)-Id
104	Beta Lactam	cphA_3	Aminoglycoside	AAC(6')-Ib_1
105	Beta Lactam	blaZ	Aminoglycoside	ANT(4')
106	Beta Lactam	blaVEB	Aminoglycoside	APH(3")-Ib
107	Beta Lactam	blaCTX-M-8	Aminoglycoside	AAC(6')-Iv_Ih
108	Beta Lactam	blaB	Aminoglycoside	APH(3')-VIIIa
109	Beta Lactam	blaIND	Aminoglycoside	AAC(6')-Ig
110	Beta Lactam	blaGES	Aminoglycoside	APH(3')-VIa
111	Beta Lactam	bla1	Aminoglycoside	aadA5_2
112	Beta Lactam	blaTLA	Aminoglycoside	AAC(6')-Ie-APH(2")-Ia
113	MDR	oprD	Aminoglycoside	AAC(6')-Is_Iu_Ix
114	MDR	mdtA	Aminoglycoside	AAC(6')-Iy
115	MDR	mdtH	Aminoglycoside	AAC(6')-Ie-APH(2")-Ia_1
116	MDR	tcrB	Aminoglycoside	AAC(6')-II
117	MDR	emrD_1	Aminoglycoside	armA_1
118	MDR	qacEdelta1_4	Aminoglycoside	AAC(3)-IIc
119	MDR	mepA	Aminoglycoside	APH(9)-Ia_1
120	MDR	arsA	Aminoglycoside	AAC(6')-I
121	MDR	pbrT	Aminoglycoside	APH(3')-VIIa
122	MDR	cadC	Aminoglycoside	AAC(6')-Im
123	MDR	sugE	Aminoglycoside	AAC(6')-Ij
124	MDR	czcA	Aminoglycoside	APH(2")-Iia
125	MDR	mexB	Aminoglycoside	AAC(6')-I-43
126	MDR	ttgA	Aminoglycoside	AAC(6')-Iw

127	MDR	mdtE	Aminoglycoside	armA_2
128	MDR	tolC_2	Sulfonamide	sul2_2
129	MDR	acrF	Sulfonamide	sul1_2
130	MDR	qacA/B	Sulfonamide	sul4
131	MDR	pcoA	Sulfonamide	sul3_1
132	MDR	acrR_1	Sulfonamide	folA_1
133	MDR	copA	Sulfonamide	folP_2
134	MDR	acrA_1	Phenicol	cmlV
135	MDR	acrB_1	Phenicol	catQ
136	MDR	qacH_1	Phenicol	cmlA_2
137	MDR	oqxA	Phenicol	cmlA_4
138	MDR	mdtG_1	Phenicol	cmxA
139	MDR	terW	Phenicol	floR_1
140	MDR	adeA	Phenicol	ceoA
141	MDR	cmr	Phenicol	mdtL_1
142	MDR	mexE	Phenicol	floR
143	MDR	mexA	Phenicol	mdtL
144	MDR	adeI	Phenicol	catIII
145	MDR	mdsA	Phenicol	catB9
146	MDR	marR_3	Phenicol	catB3
147	MDR	bexA_1	Phenicol	cat
148	MDR	pmrA	Phenicol	optrA
149	MDR	mtrE	Phenicol	catB8
150	MDR	emrB/qacA_1	Phenicol	catP
151	MDR	cfr	Phenicol	catA1
152	Other	bacA	Phenicol	cat(pC221)
153	Other	qacEdelta1_3	Phenicol	catB2
154	Other	qacEdelta1_1	Phenicol	catA2
155	Other	merA	Phenicol	fexA
156	Other	SAT-4	Other	nimE
157	Other	MCR-1	Other	fosX
158	Other	fabK	Other	fosB
159	Other	arr-3	Other	MCR-2
160	Other	nisB_1	Other	crAss56
161	Other	arr-2	Other	crAss64
162	Other	ttgB		

Table S4. Baseline relative abundance and directional shifts of mobile genetic elements (MGEs).

MGE category	No. of subtypes	Baseline relative abundance (median)	Baseline IQR	Median Δ (digester − substrate)	Δ / baseline (%)	p_adj_fdr	Directional support
Integrons	4	1.56×10^{-3}	1.38×10^{-2}	-3.76×10^{-4}	-24.1	0.490	No
Plasmid-associated	12	2.92×10^{-5}	7.19×10^{-5}	8.76×10^{-7}	3.0	0.930	No
Transposon-related	32	6.75×10^{-4}	3.63×10^{-3}	-2.19×10^{-4}	-32.5	0.008	Yes
Other-MGE	4	1.01×10^{-4}	3.87×10^{-4}	-1.06×10^{-5}	-10.6	0.685	No
Overall MGEs (pooled)	52	4.68×10^{-4}	2.81×10^{-3}	-1.19×10^{-4}	-25.4	0.006	Yes

Baseline relative abundance was calculated as the median and interquartile range (IQR) across substrate mix samples. Median Δ values represent digester-level directional shifts in relative abundance (digester − substrate). Relative changes (Δ/baseline, %) are provided for contextual interpretation only and were not subjected to additional statistical testing. Directional support indicates whether the digester-level median Δ deviated from zero based on Wilcoxon signed-rank tests with FDR correction.

Table S5. Top hub nodes identified across different co-occurrence networks

Network type	Node name	Node type	Degree
Genus-ARG	ermF_1	ARG	12
Genus-ARG	AAC(3)-III	ARG	8
Genus-ARG	ANT(6)-Ia_1	ARG	8
Genus-ARG	tcrB	ARG	6
Genus-ARG	lnuB	ARG	5
Genus-ARG	sugE	ARG	5
Genus-ARG	ANT(6)-Ia	ARG	4
Genus-ARG	tet(36)_1	ARG	3
Genus-ARG	tet(44)	ARG	3
Genus-ARG	ermF	ARG	3
Genus-ARG	*Acholeplasma* [Firmicutes]	Genus	13
Genus-ARG	*Syntrophomonas* [Firmicutes]	Genus	8
Genus-ARG	*HN-HF0106* [Firmicutes]	Genus	6
Genus-ARG	*Ruminiclostridium* [Firmicutes]	Genus	6
Genus-ARG	*Sedimentibacter* [Firmicutes]	Genus	5
Genus-ARG	*Dethiobacter* [Firmicutes]	Genus	5
Genus-ARG	*Caldicoprobacter* [Firmicutes]	Genus	4
Genus-ARG	*UCG-004* [Firmicutes]	Genus	4
Genus-ARG	*Christensenellaceae R-7 group* [Firmicutes]	Genus	3
Genus-ARG	*HT002* [Firmicutes]	Genus	3
Genus-MGE	*Bacteroides* [Bacteroidota]	Genus	4
Genus-MGE	*Bifidobacterium* [Actinobacteriota]	Genus	3
Genus-MGE	*Peptostreptococcus* [Firmicutes]	Genus	2
Genus-MGE	*Caldicoprobacter* [Firmicutes]	Genus	1
Genus-MGE	*Lentimicrobium* [Bacteroidota]	Genus	1
Genus-MGE	*Corynebacterium* [Actinobacteriota]	Genus	1
Genus-MGE	*Fastidiosipila* [Firmicutes]	Genus	1
Genus-MGE	*Christensenellaceae R-7 group* [Firmicutes]	Genus	1
Genus-MGE	*Atopostipes* [Firmicutes]	Genus	1
Genus-MGE	*UCG-005* [Firmicutes]	Genus	1
Genus-MGE	tnpA_7	MGE	6
Genus-MGE	trbC	MGE	3
Genus-MGE	IS6/257	MGE	2
Genus-MGE	IncQ_oriT	MGE	2
Genus-MGE	tnpA_5	MGE	1
Genus-MGE	IS256	MGE	1
Genus-MGE	IS1111	MGE	1
Genus-MGE	IS630	MGE	1
Genus-MGE	Tp614	MGE	1
Genus-MGE	Tn5	MGE	1

ARG-MGE	tetO_2	ARG	15
ARG-MGE	blaOXY	ARG	13
ARG-MGE	APH(3')-Ib	ARG	11
ARG-MGE	oprD	ARG	8
ARG-MGE	rmtB	ARG	8
ARG-MGE	penA	ARG	7
ARG-MGE	tetD	ARG	7
ARG-MGE	ereA	ARG	7
ARG-MGE	cfxA	ARG	7
ARG-MGE	blaMIR	ARG	7
ARG-MGE	orf37-IS26	MGE	29
ARG-MGE	ISEcp1	MGE	21
ARG-MGE	IS21-ISAs29	MGE	20
ARG-MGE	IS630	MGE	19
ARG-MGE	tnpA_5	MGE	18
ARG-MGE	ISCR1	MGE	16
ARG-MGE	Tn5403	MGE	16
ARG-MGE	IS1111	MGE	15
ARG-MGE	EAE_05855	MGE	13
ARG-MGE	intI3	MGE	11

Top hub nodes identified in the co-occurrence networks among bacterial genera, antibiotic resistance genes (ARGs), and mobile genetic elements (MGEs) across 42 samples. Co-occurrence networks were constructed based on Spearman's rank correlation analysis, and only significant correlations ($|\rho| > 0.6$ and false discovery rate (FDR)–adjusted $p < 0.05$) were retained. Hub nodes were defined based on degree values, representing the number of significant correlations with other nodes within each network.

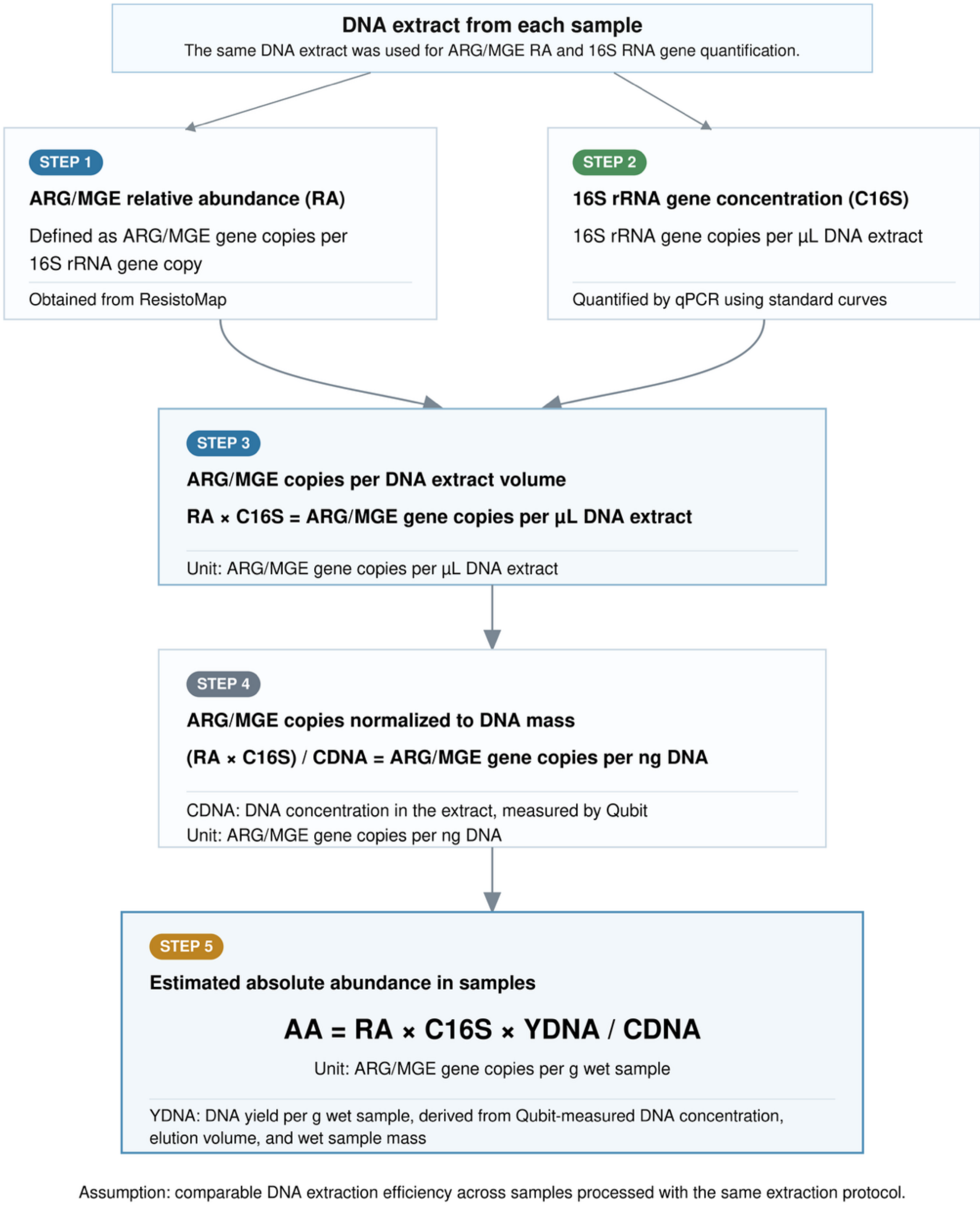

Figure S1. Calculation of ARG and MGE absolute abundance. Workflow converting relative abundance to gene copies per gram wet sample.

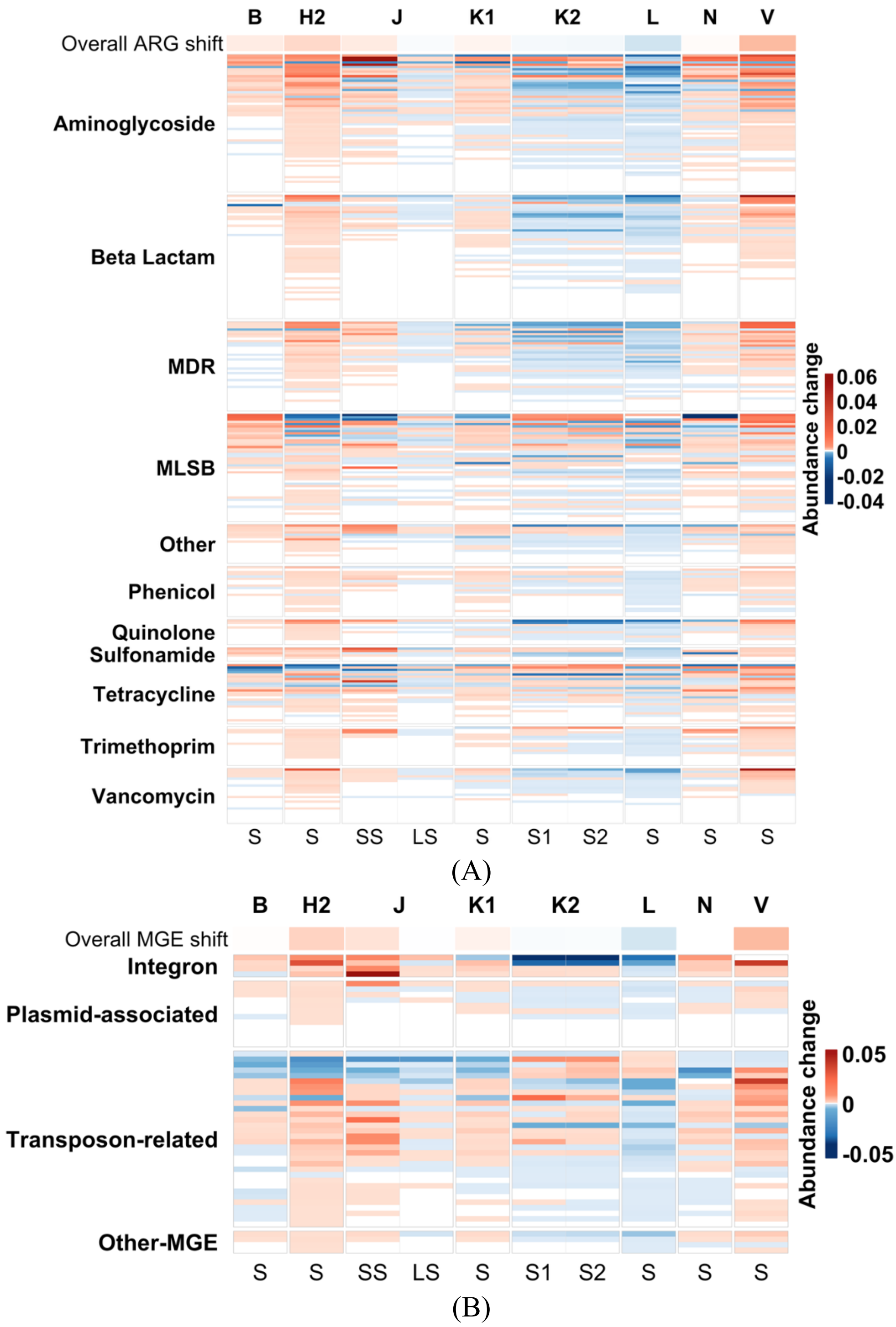

Figure S2. Fate of antibiotic resistance genes (ARGs) and mobile genetic elements (MGEs) during storage in full-scale biogas plants. Changes in relative abundance are expressed as the difference between storage digestate and the corresponding digester samples (storage − digester). Red and blue indicate increases and decreases in relative abundance, respectively. (A) ARG subtypes grouped by antibiotic classes. (B) MGEs grouped by functional categories. Columns represent individual storage systems; S, SS, and LS denote storage, solid storage, and liquid storage, respectively. For both panels, the top bar summarizes the overall median change (Δ) in relative abundance for each storage system, with blue and red indicating negative and positive deviations, respectively. The colour scale of the top bar is independent of that of the main heatmap and is used solely to indicate the direction of overall change.

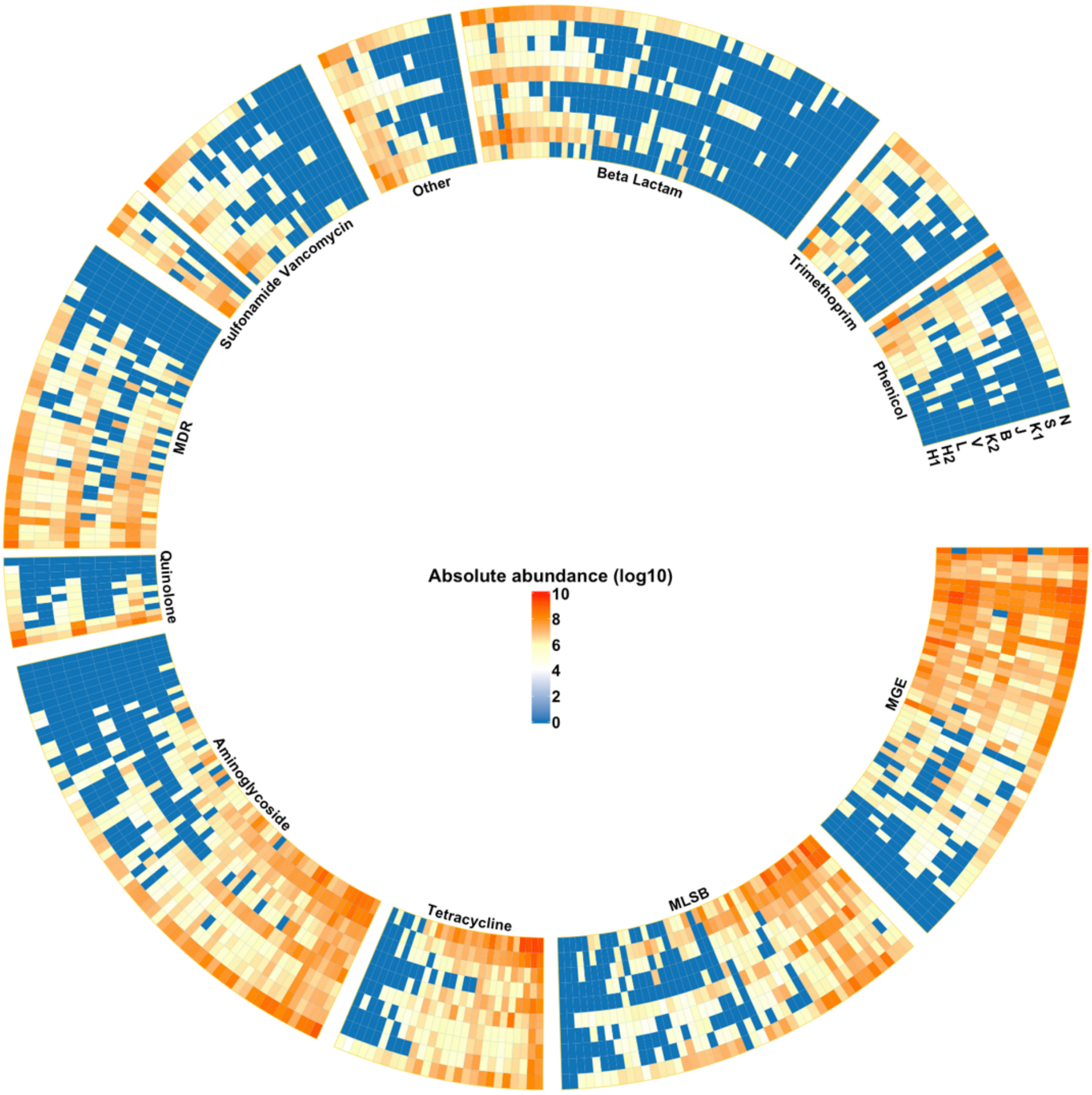

Figure S3. Absolute abundances of antibiotic resistance genes (ARGs) and mobile genetic elements (MGEs) in substrate samples from ten biogas plants. ARGs are grouped by antibiotic resistance class, with MGEs shown separately. Absolute abundances are expressed as gene copy numbers per gram of wet substrate mass and were log10-transformed for visualization. Warmer colors indicate higher abundance, whereas blue indicates low or undetected abundance.

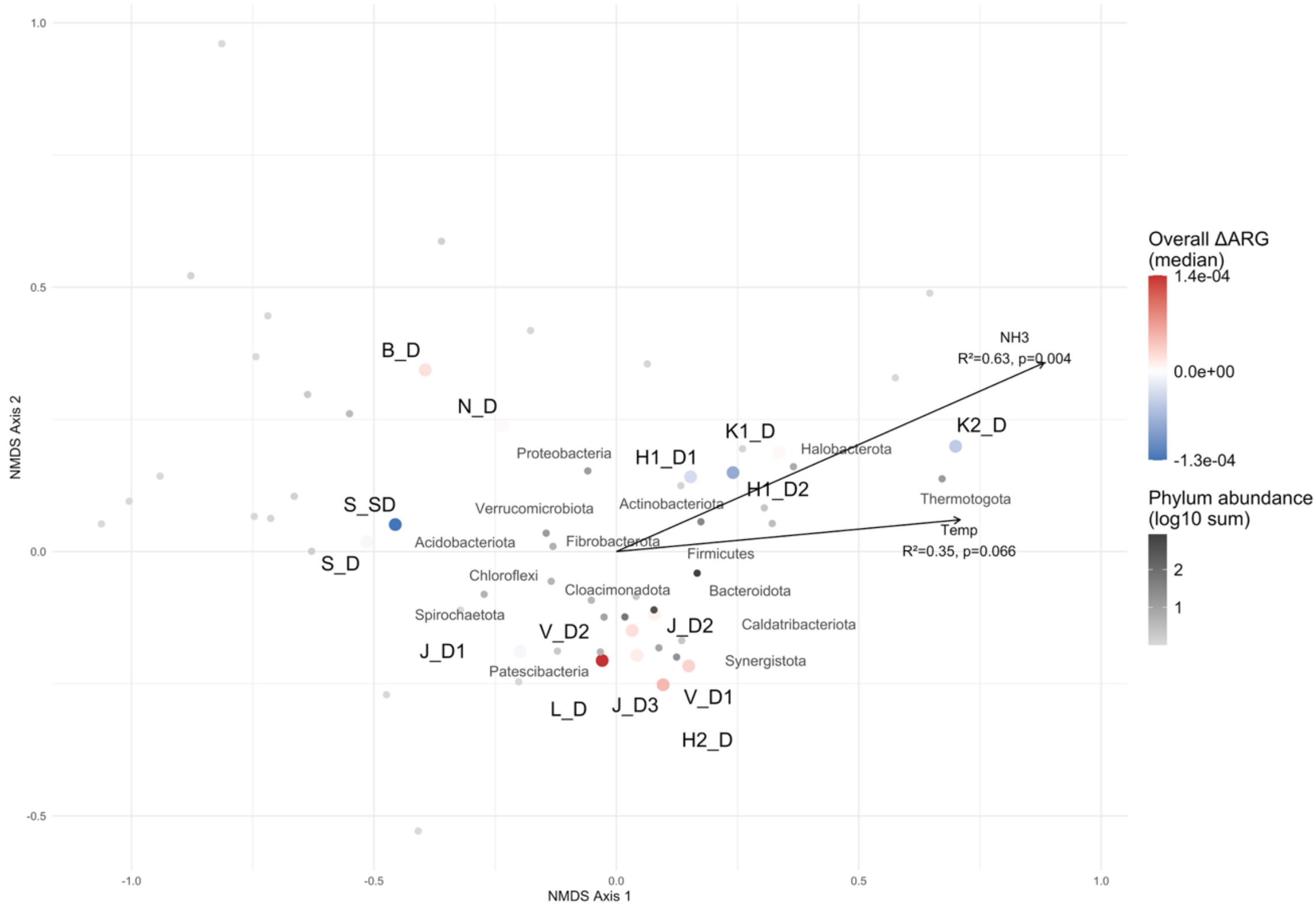

Figure S4. Non-metric multidimensional scaling (NMDS) ordination of full-scale biogas digesters based on microbial community composition at the phylum level. Digester samples are coloured according to the overall median shift in ARG relative abundance (ΔARG), calculated as the difference between digesters and their corresponding substrates (Digester − Substrate). Colour gradients range from blue (negative change) to red (positive change), with white indicating no net change. Arrows represent fitted physicochemical parameters showing significant correlations with the ordination ($p < 0.1$). Grey-scale shading of phylum labels indicates summed phylum-level abundance (log_{10}-transformed) across all digesters; only the 15 most abundant phyla are labelled to improve visual clarity.

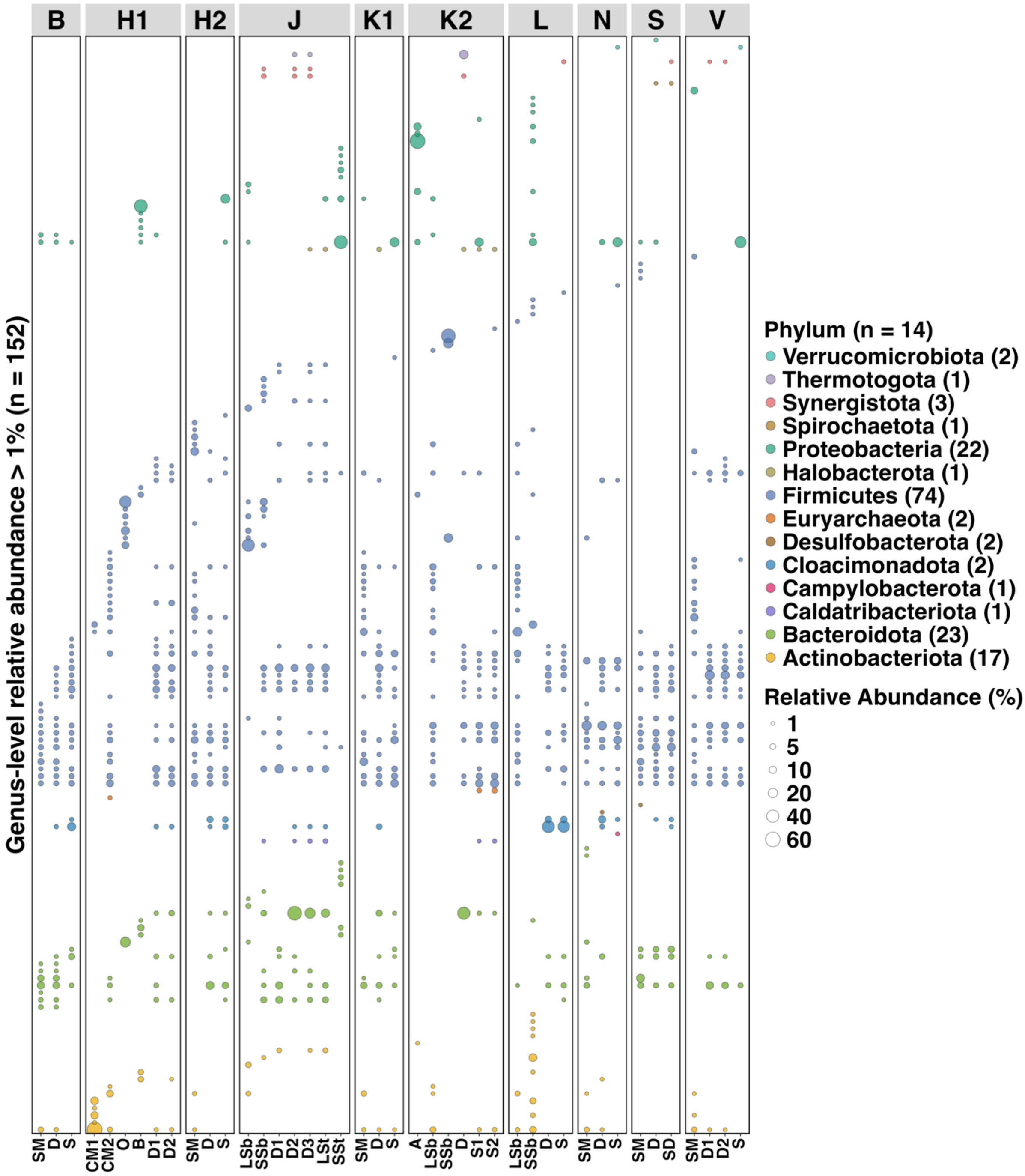

Figure S5. Genus-level bacterial community composition across substrates, digestates, and storage samples from the ten investigated biogas plants. Bubble plot showing bacterial genera with relative abundance > 1% across all samples, comprising a total of 152 genera. Bubble size corresponds to genus-level relative abundance (%), while colours indicate bacterial phyla. Numbers in parentheses denote the total number of genera detected within each phylum across all samples. Abbreviations: SM, substrate mix; D, D1, D2, digestate samples; SD, secondary digestate; S, storage sample; CM1, chicken manure; CM2, cow manure; O, oat water; B, bedding material; LSb, liquid substrate; SSb, solid substrate; A, chaff; LSt, liquid storage; SSt, solid storage.

METHODS

Physicochemical Analyses. Total solids (TS) and volatile solids (VS) were determined in triplicate using standard methods.[1] Concentrations of ammonium nitrogen (NH_4^+-N) and volatile fatty acids (VFAs), as well as pH, were measured following procedures described previously.[2] Ammonia nitrogen (NH_3-N) concentrations were calculated from temperature, pH, and NH_4^+-N according to a published approach.[3]

DNA Extraction. DNA was extracted in triplicate from all samples using the FastDNA Spin Kit for Soil (MP Biomedicals Europe) following the manufacturer's instructions. Extracted DNA was purified using AMPure magnetic beads (Beckman Coulter) following the manufacturer's protocol, and DNA concentrations were quantified using a Qubit fluorometer.

Molecular Analyses. Relative abundances (RA) of ARGs and MGEs were determined by Resistomap Oy (Helsinki, Finland) using customised SmartChip qPCR primer sets, following previously published high-throughput qPCR frameworks for ARGs and MGEs[4–6]. ARG assay naming was based on the CARD database. The panel included 323 ARG subtypes and 52 MGE markers (Table S3). Gene detection required appropriate melting curves, no primer-dimer signals, Cq <28, and qPCR efficiency within 2 ± 0.3. RA values were normalised to 16S rRNA gene abundance using the $2^{-\Delta Cq}$ method.

Absolute abundances (AA) of total bacteria were quantified by qPCR targeting the 16S rRNA gene using primers Bac338F (5′-ACTCCTACGGGAGGCAGCAG-3′)[7] and Bac534R (5′-ATTACCGCGGCTGCTGGC-3′)[8] on a QuantStudio™ 5 Real-Time PCR System with SYBR Green chemistry. Each reaction (20 µL) contained 10 µL SYBR Green master mix, 1 µL of each primer, 5 µL nuclease-free water, and 3 µL template DNA. The qPCR program consisted of 95 °C for 7 min, followed by 40 cycles of 95 °C for 40 s, 58 °C for 60 s, and 72 °C for 40 s, with melt curve analysis performed from 55 to 95 °C. All reactions were performed in technical triplicate, and template DNA was diluted as necessary to minimise PCR inhibition. Standard curves showed $R^2 > 0.99$ and amplification efficiencies between 90% and 110%. Microbial community composition was characterised by 16S rRNA gene amplicon sequencing (Illumina platform) performed by Novogene (Netherlands), following a previously described protocol.[2]

REFERENCES

(1) APHA. Standard Methods for Examination of Water and Wastewater. *American Public Health Association* **1998**, *9*.

(2) Perman, E.; Westerholm, M.; Liu, T.; Schnürer, A. Comparative Study of High-Solid Anaerobic Digestion at Laboratory and Industrial Scale – Process Performance and Microbial Community Structure. *Energy Conversion and Management* **2024**, *300*, 117978. https://doi.org/10.1016/j.enconman.2023.117978.

(3) Calli, B.; Mertoglu, B.; Inanc, B.; Yenigun, O. Effects of High Free Ammonia Concentrations on the Performances of Anaerobic Bioreactors. *Process Biochemistry* **2005**, *40* (3–4), 1285–1292. https://doi.org/10.1016/j.procbio.2004.05.008.

(4) Zhu, Y. G.; Johnson, T. A.; Su, J. Q.; Qiao, M.; Guo, G. X.; Stedtfeld, R. D.; Hashsham, S. A.; Tiedje, J. M. Diverse and Abundant Antibiotic Resistance Genes in Chinese Swine Farms. *Proceedings of the National Academy of Sciences of the United States of America* **2013**, *110* (9), 3435–3440. https://doi.org/10.1073/PNAS.1222743110/SUPPL_FILE/ST06.DOCX.

(5) Muziasari, W. I.; Pärnänen, K.; Johnson, T. A.; Lyra, C.; Karkman, A.; Stedtfeld, R. D.; Tamminen, M.; Tiedje, J. M.; Virta, M. Aquaculture Changes the Profile of Antibiotic Resistance and Mobile Genetic Element Associated Genes in Baltic Sea Sediments. *FEMS Microbiol Ecol* **2016**, *92* (4), fiw052. https://doi.org/10.1093/femsec/fiw052.

(6) Stedtfeld, R. D.; Guo, X.; Stedtfeld, T. M.; Sheng, H.; Williams, M. R.; Hauschild, K.; Gunturu, S.; Tift, L.; Wang, F.; Howe, A.; Chai, B.; Yin, D.; Cole, J. R.; Tiedje, J. M.; Hashsham, S. A. Primer Set 2.0 for Highly Parallel qPCR Array Targeting Antibiotic Resistance Genes and Mobile Genetic Elements. *FEMS Microbiol Ecol* **2018**, *94* (9), fiy130. https://doi.org/10.1093/femsec/fiy130.

(7) Dethlefsen, L.; Huse, S.; Sogin, M. L.; Relman, D. A. The Pervasive Effects of an Antibiotic on the Human Gut Microbiota, as Revealed by Deep 16S rRNA Sequencing. *PLOS Biology* **2008**, *6* (11), e280. https://doi.org/10.1371/journal.pbio.0060280.

(8) Hartman, A. L.; Lough, D. M.; Barupal, D. K.; Fiehn, O.; Fishbein, T.; Zasloff, M.; Eisen, J. A. Human Gut Microbiome Adopts an Alternative State Following Small Bowel Transplantation. *Proceedings of the National Academy of Sciences* **2009**, *106* (40), 17187–17192. https://doi.org/10.1073/pnas.0904847106.
